# Conformational preferences of cationic β-peptide in water studied by CCSD(T), MP2, and DFT methods

**DOI:** 10.1016/j.heliyon.2020.e04721

**Published:** 2020-08-14

**Authors:** Young Kee Kang, Hae Sook Park

**Affiliations:** a Department of Chemistry, Chungbuk National University, Cheongju, Chungbuk 28644, Republic of Korea; b Department of Nursing, Cheju Halla University, Cheju 63092, Republic of Korea

**Keywords:** Theoretical chemistry, Bioinformatics, Nylon-3 dipeptide, Conformational preferences, CCSD(T)/CBS-limit, MP2, DFT-D, Solvation effect

## Abstract

The conformational preferences of the cationic nylon-3 βNM [(3*R*,4)-diaminobutanoic acid, dAba] dipeptide in water were explored as the first step to understand the mode of action of polymers of βNM against phylogenetically diverse and intrinsically drug-resistant pathogenic fungi. The CCSD(T), MP2, M06-2X, *ω*B97X-D, B2PLYP-D3BJ, and DSD-PBEP86-D3BJ levels of theory with various basis sets were assessed for relative energies of the 45 local minima of the cationic Ac-dAba-NHMe located at the SMD M06-2X/6-31+G(d) level of theory in water against the benchmark CCSD(T)/CBS-limit energies in water. The best performance was obtained at the double-hybrid DSD-PBEP86-D3BJ/def2-QZVP level of theory with RMSD = 0.12 kcal/mol in water. The M06-2X/def2-QZVP level of theory predicted reasonably the conformational preference with RMSD = 0.38 kcal/mol in water and may be an alternative level of theory with marginal deviations for the calculation of conformational energies of relatively longer cationic peptides in water. In particular, the *H*_14_–helical structures appeared to be the most feasible conformations for the cationic Ac-dAba-NHMe populated at 48–64% by relative free energies in water. The hexamer built from the *H*_14_–structure of the cationic Ac-dAba-NHMe adopted a left-handed 3_14_-helix, which has a slightly narrower radius and a longer rise than the regular 3_14_-helix of β-peptides. Hence, the 3_14_-helices of oligomers or polymers of the cationic dAba residues are expected to be the active conformation to exhibit the ability to bridge between charged lipid head groups that might cause a local depression or invagination of the membrane of fungi.

## 1 Introduction

For two decades, the density functional theory (DFT) has been an efficient computational tool in conformational study of amino acids and peptides [1, 2, 3, 4, 5, 6]. Compared to correlated wave function theories, DFT has achieved a better performance. However, further investigation on higher levels of theory with electron correlations and large basis sets are necessary in order to describe non-covalent interactions in peptides.

Multiple attempts have been made using the complete basis set (CBS) extrapolation and CCSD(T) corrections to reproduce accurately (~1 kcal/mol) experimental thermochemical data, barrier heights of chemical reactions, and non-covalent interaction energies [3, 7]. However, the computational cost was a limiting factor when applying CCSD(T)/CBS methods to conformational study of amino acids [8, 9, 10] and small peptides [11, 12, 13, 14, 15]. It has been known that typical DFT correlation functionals are designed for the description of short-range correlations, whereas MP2 is superior to DFTs in the description of long-range correlations. To handle both types of correlations more appropriately, several double-hybrid DFT methods have been proposed, based on a combination of density functionals for exchange and correlation with Hartree-Fock (HF) exchange and a perturbative second-order correlation obtained from Kohn-Sham orbitals [16, 17, 18]. In particular, the addition of dispersion corrections has shown improved performance of DFTs when predicting the relative conformational energies of neutral amino acids and peptides against the benchmark CCSD(T)/CBS-limit energies [9, 10, 12, 13, 14, 15, 19]. However, no assessment of DFTs has been made for relative conformational energies of charged amino acids or peptides in water, although there was an assessment of DFTs for the clusters of the zwitterionic arginine with halide ion (Cl^−^ and Br^−^) against the benchmark MP2 energies in the gas phase [20].

**Figure 1:**
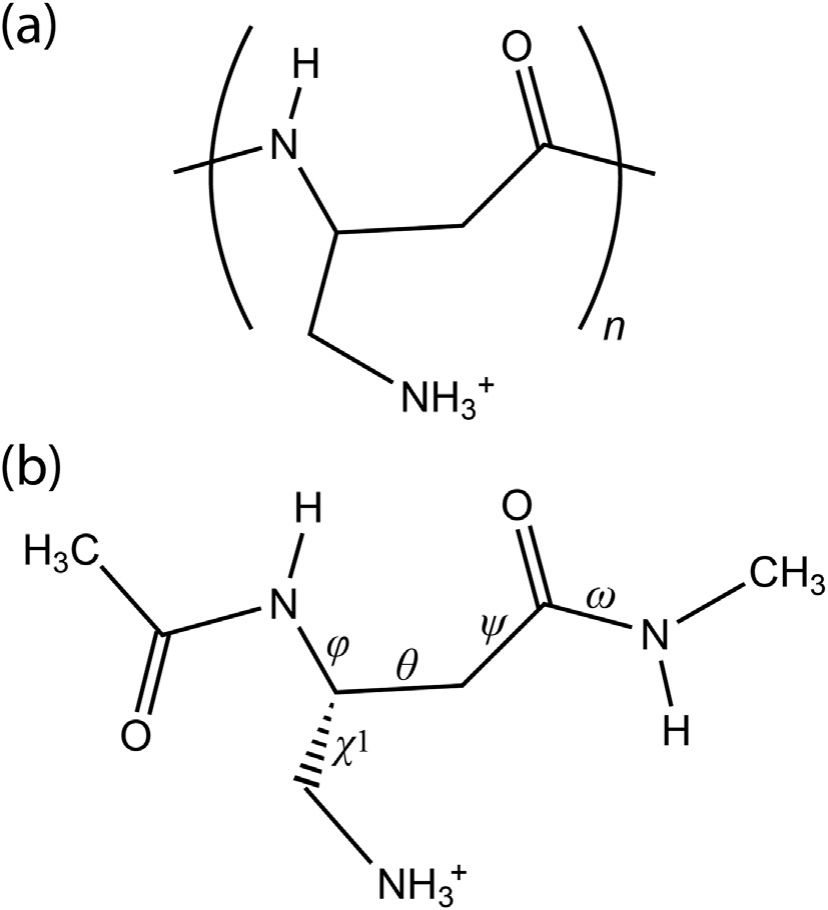
(a) Chemical structure of the cationic βNM (dAba) polymer and (b) chemical structure and torsion angles of the cationic Ac-dAba-NHMe.

Nylon-3 polymers (poly-β-peptides) are one of non-natural analogs of host-defense peptides that are produced during the innate immune response to infections by microbes such as bacteria and fungi [21]. In particular, polymers of nylon-3 βNM (Figure 1a) prepared from β-aminomethyl-β-lactam displayed activity against phylogenetically diverse, intrinsically drug-resistant pathogenic fungi with relatively low toxicity toward mammalian cells [22, 23, 24, 25]. Based upon the membrane leakage experiments by cationic βNM polymers, it was suggested that the ability of cationic polymers to bridge between charged lipid head groups might cause a local depression or invagination of the membrane of fungi [26]. However, conformational preferences of oligomers or polymers for cationic βNM studied in water studied by spectroscopic or computational methods are not available yet.

Here, we explore the conformational preferences of the cationic βNM dipeptide in water as the first step to understand the mode of action of poly-βNM. Dispersion-corrected DFTs were assessed for the relative energies of the 45 local minima of the cationic βNM dipeptide located in water against the benchmark CCSD(T)/CBS-limit energies.

## 2 Computational methods

Chemical structure and torsional parameters for the cationic βNM [(3*R*,4)-diaminobutanoic acid, abbreviated as dAba hereafter] dipeptide (Ac-dAba-NHMe) are defined in Figure 1b. The initial structure of the cationic dAba dipeptide with the extended backbone was constructed using the GaussView program [27]. All calculations were carried out using the Gaussian 09 package [28]. All geometry optimizations in water were carried out using the M06-2X functional [29] and the Solvation Model based on Density (SMD) method [30] with the “fine” integration grid that is the default in the Gaussian 09 package. Although the M06-2X functional is a hybrid-meta-GGA functional with the improved medium-range correlation energy, it have been known to exhibit good performance in predicting noncovalent interactions of small molecules and relative stabilities of biological compounds [4]. In particular, the SMD M06-2X/6-31+G(d) level of theory well reproduced solvation free energies of model compounds for the backbone and side chains of peptides in water [31].

Using the extended structure of the neutral and cationic Ac-dAba-NHMe, 1,260 (i.e., 644 and 616 structures for neutral and cationic dipeptides, respectively) initial structures were generated by the systematic search of the Discovery Studio package [32] using the CHARMm force field with the maximum systematic conformations = 1000 and the energy threshold = 20 kcal/mol in the gas phase. In the conformational search, a systematic variation of each of the torsion angles *φ*, *θ*, *ψ* of the backbone and *χ*^1^ of the side chain was done using steps of 60°. The initial neutral structures were edited by adding protons to produce the cationic species. These 1,260 initial structures were optimized at the SMD HF/3-21G(d) level of theory in water and we obtained 58 local minima with relative energy (Δ*E*) < 10 kcal/mol, which were reoptimized at the SMD M06-2X/6-31G(d) level of theory in water and further optimized at the SMD M06-2X/6-31+G(d) level of theory in water. Finally, we obtained the 45 local minima for the cationic Ac-dAba-NHMe with Δ*E* < 5 kcal/mol in water.

Vibrational frequencies were calculated for the 45 local minima for the cationic dAba dipeptide at the SMD M06-2X/6-31+G(d) level of theory in water at 25 °C and 1 atm, of which the scale factor was 0.9440 to reproduce experimental frequency of 1707 cm^−1^ for the amide I band of *N*-methylacetamide in Ar and N_2_ matrixes [33]. The Gibbs free energy of each conformation was calculated from the zero-point energy correction and the thermal energy corrections, from which the populations of all local minima were estimated at 25 °C in water. The translational, rotational, and vibrational contributions to the Gibbs free energy were computed using the ideal gas, rigid rotor, and harmonic oscillator approximations, respectively [34].

Single-point energies (Δ*E*_sp_) of the 45 local minima for the cationic dAba dipeptide were calculated at the MP2 level of theory with cc-pVDZ and aug-cc-pVXZ (X = D, T, and Q) basis sets and the CCSD(T) level of theory with the cc-pVDZ basis set. Each CCSD(T)/CBS-limit energy was estimated by the sum of the MP2/CBS-limit energy and the “coupled-cluster correction”, ΔCCSD(T). The MP2/CBS-limit energy was obtained using the two-point extrapolation scheme of Halkier et al. [35] with MP2/aug-cc-pVTZ and MP2/aug-cc-pVQZ energies. The value of ΔCCSD(T) was calculated by the difference between CCSD(T) and MP2 energies with the cc-pVDZ basis set. Here, the cc-pVDZ, aug-cc-pVDZ, aug-cc-pVTZ, and aug-cc-pVQZ basis sets are abbreviated as DZ, aDZ, aTZ, and aQZ, respectively.

Then, we assessed the four DFTs classified by rung 4 and rung 5 on the Perdew's “Jacob's Ladder” [36, 37] for relative energies of the 45 local minima for the cationic dAba dipeptide against the benchmark CCSD(T)/CBS-limit energies: two M06-2X [29] and *ω*B97X-D [38] functionals; and two double-hybrid B2PLYP [39] and DSD-PBEP86 [40] functionals. The basis sets used are cc-pVTZ, def2-TZVP, and def2-QZVP, which are abbreviated as TZ, dTZ, and dQZ, respectively. In particular, the corrections of dispersion were calculated using the Grimme's D3 version with Becke-Johnson damping (i.e., D3BJ) [41] for double-hybrid B2PLYP and DSD-PBEP86 functionals. Recently, the DSD-PBEP86-D3BJ and B2PLYP-D3BJ levels of theory exhibited the best performance against the relative CCSD(T)/CBS-limit energies for the Ala and Pro dipeptides and the Ala tetrapeptide, whereas the M06-2X and *ω*B97X-D functionals were suggested as an alternative level of theory with marginal deviations [9, 15].

The helix parameters of the *H*_14_ hexamer were calculated from its six consecutive β-carbons using the HELFIT program [42], based on total least squares algorithm for helix fitting with at least four data points for the analysis.

## 3 Results and discussion

### 3.1 Conformational preference of the cationic Ac-dAba-NHMe in water

Torsion angles, relative electronic energies, and solvation free energies of the 45 local minima for the cationic Ac-dAba-NHMe in water located at the CCSD(T)/CBS-limit//SMD M06-2X/6-31+G(d) level of theory are listed in Table 1. For comparison, the conformational codes for Ac-βAbu-NHMe with the –CH^β^(CH_3_)–CH_2_^α^– backbone obtained at the SCRF HF/6-31G(d) level of theory [43] are shown in parentheses. For C=O⋯H–N H-bonds, the maximum value of the O⋯H distance and the minimum value of the O⋯H–N angle were taken as 2.4 Å and 120°, respectively [44]. There were several types of H-bond for the cationic Ac-dAba-NHMe. *C*_6_ and *C*_8_ are 6- and 8-membered H-bonds for backbone. *C*_7a_ (or *C*_7b_) is the 7-membered H-bond of the H–N^+^ of the side chain of the dAba residue with the C=O of the acetyl group (or the dAba residue). *H*_14_, *H*_10/12_, and *H*_10_ are the monomer of helices with 14-, 10/12-, and 10-membered H-bonded turns, respectively, which are available for oligomers beyond the monomer or dimer (see Ref. [43]). Absolute and relative thermodynamic properties and solvation free energies of the 45 local minima of the cationic Ac-dAba-NHMe with Δ*E* < 5 kcal/mol optimized at the SMD M06-2X/6-31+G(d) level of theory in water are listed in Table S1 of the Supplementary material. In addition, absolute and relative single-point energies of the same 45 local minima calculated by M06-2X, *ω*B97X-D, B2PLYP-D3BJ, DSD-PBEP86-D3BJ, MP2, and CCSD(T) methods are shown in Tables S2–S5 of the Supplementary material. Cartesian coordinates of the 45 local minima optimized at the SMD M06-2X/6-31+G(d) level of theory in water are also listed in Table S6 of the Supplementary material.

**Table 1:** Torsion angles, relative electronic energies, and solvation free energies of the 45 local minima of the cationic Ac-dAba-NHMe with Δ*E* < 5 kcal/mol at the CCSD(T)/CBS limit//SMD M06-2X/6-31+G(d) level of theory in water.^a^

Conf.^b^	Type^c^	*φ*	*θ*	*ψ*	*χ*^1^	Δ*E*_sp_^d^	Δ*G*_s_^e^	Δ*E*^f^
m01 (B17^s^)	*C*_7a_/*C*_7b_	70.3	177.9	-105.4	-179.9	0.00	-65.56	0.00
m02 (B10^s^)	*H*_14_	-162.2	61.7	-137.2	-176.2	13.65	-78.73	0.48
m03 (B10^s^)	*H*_14_	-142.7	62.9	-140.5	-177.1	19.13	-83.95	0.75
m04	*C*_6_/*C*_7a_	-173.3	54.1	102.2	-178.7	10.15	-74.86	0.85
m05	*C*_7a_	65.4	59.2	-131.4	-177.9	11.17	-75.87	0.87
m06 (B10^s^)	*H*_14_	-124.4	60.6	-137.7	-176.6	24.40	-89.07	0.90
m07 (B1^s^)	*C*_6_	-143.9	-62.6	-127.7	-177.8	13.43	-77.94	1.05
m08	*C*_7b_	-155.2	-171.8	-103.8	179.5	13.18	-77.60	1.15
m09	*C*_7b_	-155.8	-53.8	147.6	176.8	8.13	-72.36	1.33
m10 (B3^s^)	*C*_7b_	-80.0	-75.4	107.3	-179.9	14.64	-78.81	1.39
m11 (B16^s^)		-147.8	173.0	131.2	178.0	17.70	-81.71	1.54
m12	*C*_7b_	-75.0	155.0	-123.3	-179.0	11.40	-75.42	1.55
m13 (B8^s^)	*C*_7a_	65.4	167.7	134.8	179.5	12.45	-76.28	1.72
m14 (B9^s^)	*H*_10/12_	-88.6	57.9	91.0	-171.9	28.45	-92.26	1.76
m15	*C*_7a_/*C*_7b_	76.4	-55.4	92.9	-170.3	5.76	-69.47	1.85
m16 (B16^s^)	*C*_7a_/*C*_7b_	-172.4	173.0	171.3	177.5	3.73	-67.42	1.87
m17 (B10^s^)	*H*_14_	-155.3	60.9	-133.7	-177.2	28.21	-91.88	1.89
m18 (B6^s^)	*C*_8_	-116.7	65.8	10.2	178.9	29.82	-93.45	1.93
m19 (B19^s^)	*C*_7b_	-151.4	-78.7	111.2	178.8	13.66	-77.27	1.96
m20	*C*_7b_	-81.8	-173.7	-99.6	178.4	16.98	-80.55	1.99
m21 (B18^s^)	*H*_10_	59.7	52.0	86.7	-177.8	14.53	-78.09	2.00
m22 (B3^s^)	*C*_7b_	-64.4	-38.6	112.6	-177.7	13.15	-76.69	2.02
m23	*C*_6_/*C*_7b_	-156.1	-61.8	-179.1	-179.5	8.74	-72.27	2.03
m24 (B1^s^)	*C*_6_	-151.8	-63.7	-128.6	-176.8	25.11	-88.62	2.05
m25	*H*_14_	-139.0	59.5	-133.9	-179.0	30.24	-93.71	2.09
m26	*C*_7a_	-166.0	167.4	-101.1	179.0	11.30	-74.57	2.29
m27		61.8	55.2	-132.0	-179.4	21.36	-84.41	2.51
m28		-160.5	178.2	127.8	177.9	29.49	-92.48	2.57
m29		-142.7	63.2	14.5	179.3	24.23	-87.21	2.59
m30 (B9^s^)	*H*_10/12_	-88.2	56.6	92.0	-173.5	34.15	-96.90	2.81
m31	*C*_7b_	68.5	-79.6	123.1	-176.6	12.16	-74.85	2.87
m32	*C*_7b_	69.2	164.1	-155.3	-177.5	9.36	-71.87	3.05
m33	*C*_6_	-160.1	58.3	103.0	-179.8	26.80	-89.30	3.06
m34	*C*_6_	-165.3	58.3	98.5	-177.2	22.58	-85.02	3.13
m35 (B6^s^)	*C*_8_	-113.8	62.7	17.1	179.1	32.43	-94.71	3.28
m36		63.6	168.5	128.6	178.7	22.63	-84.91	3.29
m37		-77.6	170.6	125.2	179.6	38.00	-100.08	3.49
m38 (B6^s^)	*C*_8_	-114.3	69.5	6.2	-179.6	34.62	-96.67	3.51
m39		67.5	56.3	-133.2	-178.6	25.05	-87.09	3.52
m40 (B18^s^)	*H*_10_	59.1	48.9	85.6	-176.6	27.14	-89.14	3.56
m41 (B3′^s^)		56.4	47.0	-113.5	177.3	25.99	-87.88	3.67
m42		-149.9	66.6	7.6	179.9	32.27	-94.06	3.78
m43	*C*_7a_	79.6	-57.6	93.2	-169.9	21.63	-83.29	3.90
m44 (B2^s^)	*C*_8_	-71.4	139.7	-74.8	175.0	29.88	-91.32	4.12
m45 (B12^s^)	*C*_7a_	-173.7	-70.7	18.4	178.4	20.43	-81.22	4.77

a Units for torsion angles, relative electronic energies, and solvation free energies in degrees, kcal/mol, and kcal/mol, respectively. Torsion angles are defined in Figure 1b. All local minima were optimized at the SMD M06-2X/6-31+G(d) level of theory in water.

b The conformation in parentheses were for Ac-βAbu-NHMe from Table V of Ref. [43].

c Conformation types. *C*_6_ and *C*_8_ are 6- and 8-membered H-bonds for backbone. *C*_7a_ and *C*_7b_ are 7-membered H-bonds between the C=O of the acetyl group and the H–N^+^ of the side chain and between the C=O of the dAba residue and the H–N^+^ of the side chain, respectively. *H*_*n*_ is the monomer of helix with *n*-membered H-bonded turn available for oligomers beyond the monomer or dimer.

d The relative CCSD(T)/CBS limit single-point energies.

e Solvation free energies were calculated at the SMD M06-2X/6-31+G(d) level of theory in water.

f Each relative energy in water was calculated by the sum of the Δ*E*_sp_ and Δ*G*_s_ energies.

**Figure 2:**
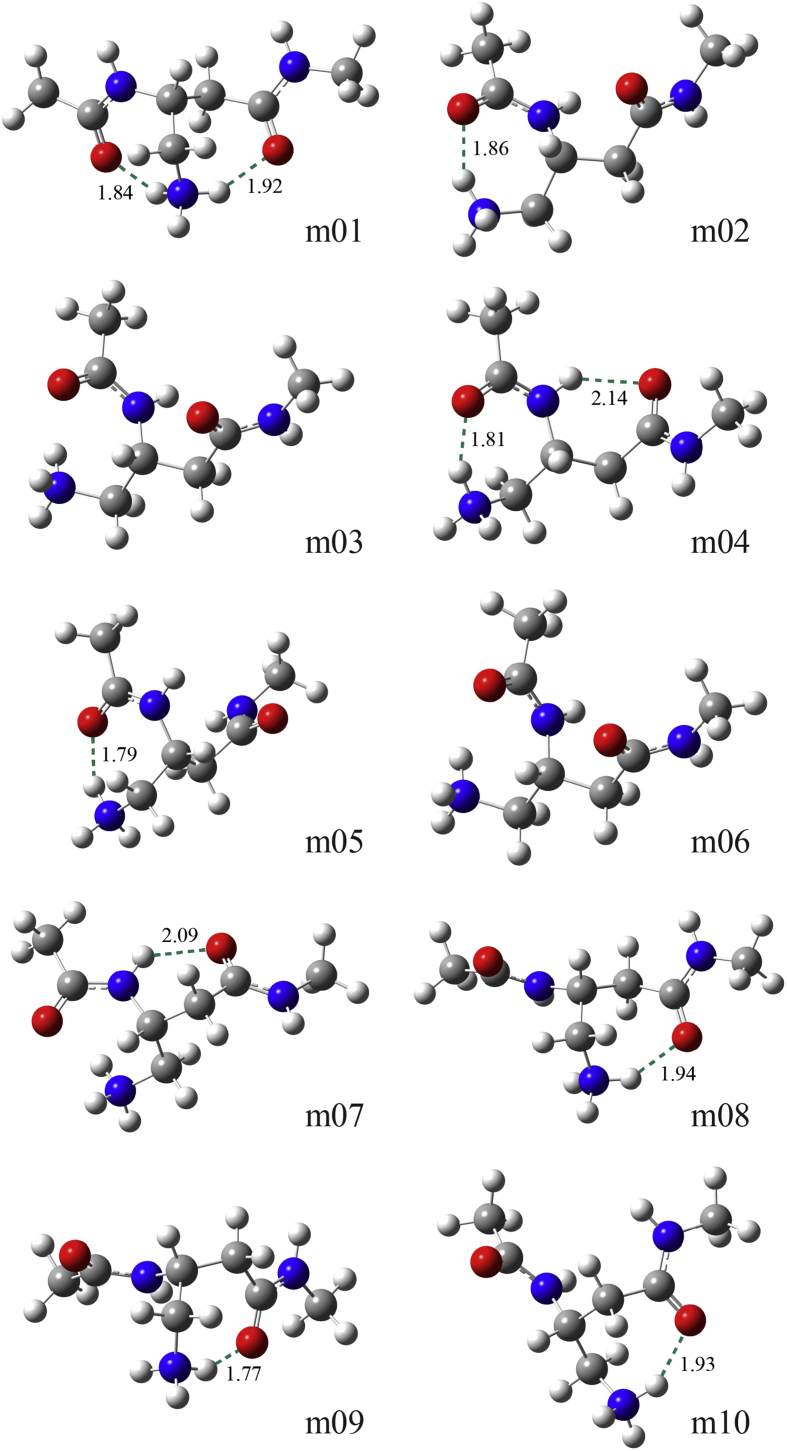
Structures of the first ten lowest-energy conformations for the cationic Ac-dAba-NHMe with Δ*E* < 1.50 kcal/mol in water obtained at the CCSD(T)/CBS limit//SMD M06-2X/6-31+G(d) level of theory. H-bonds are represented by dotted lines and their distances are in Å.

The optimized structures of the first ten lowest-energy conformers (m01−m10) with Δ*E* < 1.5 kcal/mol in water at the CCSD(T)/CBS-limit//SMD M06-2X/6-31+G(d) level of theory are shown in Figure 2. The lowest-energy conformer m01 was stabilized by a bifurcated H-bond of the H–N^+^ group of the side chain with two C=O groups of the backbone with *d*(C=O⋯H–N^+^) = 1.84 and 1.92 Å for *C*_7a_ and *C*_7b_ H-bonds, respectively. The second and third lowest-energy conformers (m02 and m03) adopted the *H*_14_ conformation similar to the monomer of a helix with 14-membered H-bonded turns. Although conformers m02 and m03 had similar relative energies (Δ*E* = 0.48 and 0.75 kcal/mol, respectively), the former had the favored single-point energy (Δ*E*_sp_) by 5.48 kcal/mol but the disfavored solvation energy (Δ*G*_s_) by 5.22 kcal/mol than the latter. This seemed to be due to the formation of a *C*_7a_ H-bond for the former between the acetyl C=O group and the cationic H–N^+^ group of the side chain with *d*(C=O⋯H–N^+^) = 1.86 Å, whereas the latter had the favored solvation due to the longer distance between *C*_7a_ H-bond partners with *d*(C=O⋯H–N^+^) = 2.59 Å.

Although the following three lowest-energy conformers (m04, m05, and m06) had comparable conformational energies (0.85, 0.87, and 0.90 kcal/mol, respectively), they had different patterns of conformational features. Conformer m04 was stabilized by a *C*_6_ H-bond between the N–H group and the C=O group of the dAba residue with *d*(C=O⋯H–N) = 2.14 Å and a *C*_7a_ H-bond with *d*(C=O⋯H–N^+^) = 1.81 Å. Conformer m05 was stabilized by a short *C*_7a_ H-bond with *d*(C=O⋯H–N^+^) = 1.79 Å. Although conformer m06 adopted a *H*_14_ conformation similar to conformer m02 and m03, the former was 10.75 and 5.27 kcal/mol less favored in Δ*E*_sp_ but −10.34 and −5.12 kcal/mol more favored in Δ*G*_s_ than the latter, respectively.

The Δ*E* values of the next four lowest-energy conformers (m07−m10) were 1.05–1.39 kcal/mol, in which conformer m07 had a *C*_6_ H-bond with the distance of *d*(C=O⋯H–N) = 2.09 Å, whereas conformers m08−m10 had a *C*_7b_ H-bond with *d*(C=O⋯H–N^+^) = 1.94, 1.77, and 1.93 Å, respectively. In particular, conformers m15 and m16 with a bifurcated H-bond with *C*_7a_ and *C*_7b_ types of H-bonds, as in the lowest-energy conformer m01, had the third and second lowest value of Δ*E*_sp_ = 5.76 and 3.73 kcal/mol, respectively, but its Δ*E* value was 1.85 and 1.87 kcal/mol in water, respectively, due to the less favored solvation. There were 10 local minima for 1.5 kcal/mol < Δ*E* < 2.0 kcal/mol; 11 local minima for 2.0 kcal/mol ≤ Δ*E* < 3.0 kcal/mol; 14 local minima for 3.0 kcal/mol < Δ*E* < 5.0 kcal/mol.

Möhle et al. explored the conformational preference of Ac-βAbu-NHMe at the SCRF HF/6-31G(d) level of theory in water [43]. The authors found the energetic stability of three representative conformers was in the order B1^s^ > B10^s^ > B17^s^ with Δ*E* = 0.00, 2.10, and 4.25 kcal/mol in water, respectively. Conformer B1^s^, B10^s^, and B17^s^ correspond to conformer m07, m02/m03, and m01 in this work, respectively (see Table 1). However, the conformational preference for the cationic Ac-dAba-NHMe were calculated as in the order m01 > m02 > m03 > m07 with Δ*E* = 0.00, 0.48, 0.75, and 1.05 kcal/mol in water, respectively, at the SMD M06-2X/6-31+G(d) level of theory. As described above, conformer m01 had a bifurcated H-bond of the H–N^+^ group of the side chain with two C=O groups of the backbone, conformer m02 and m03 adopted a *H*_14_-type structure, and conformer m07 had a *C*_6_ H-bond. The different conformational preference of the cationic Ac-dAba-NHMe from that of Ac-βAbu-NHMe can be ascribed to the formation of stronger H-bonds or favored electrostatic interactions between the C=O groups of the backbone and the H–N^+^ group of the side chain than the *C*_6_ H-bond between the N–H group and the C=O group of the dAba residue (see Figure 2).

### 3.2 Assessments for conformational energies in water

**Figure 3:**
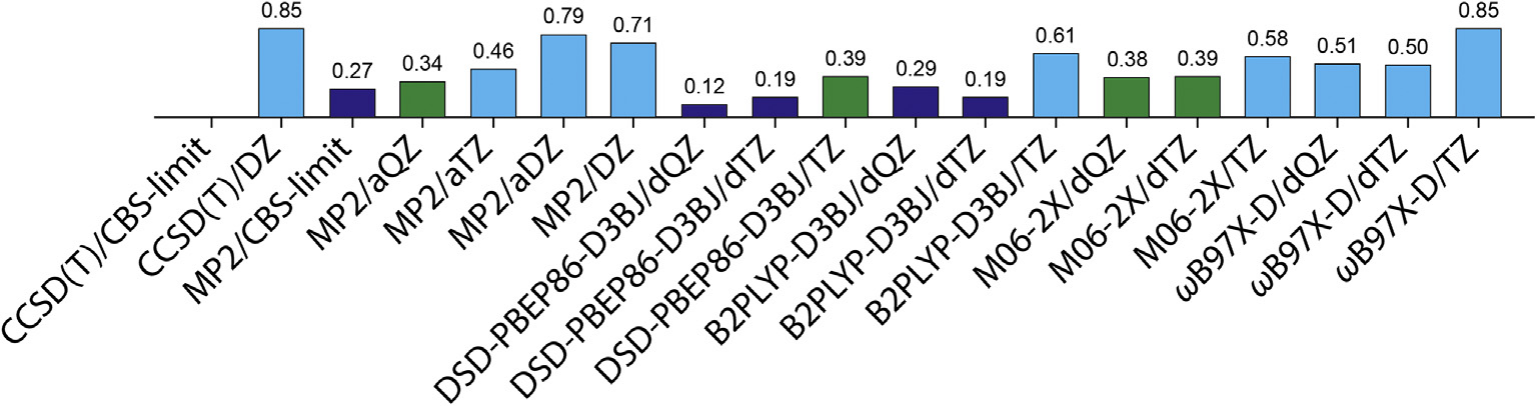
RMSDs (kcal/mol) for relative energies (Δ*E*) of the 45 local minima of the cationic Ac-dAba-NHMe in water obtained at the CCSD(T), MP2, DSD-PBEP86-D3BJ, B2PLYP-D3BJ, M06-2X, and *ω*B97X-D levels of theory with various basis sets against the benchmark CCSD(T)/CBS-limit energies. Dark blue histograms for RMSD <0.3 kcal/mol; dark green histograms for 0.3 kcal/mol ≤ RMSD ≤0.4 kcal/mol; light blue histograms for RMSD >0.4 kcal/mol.

The relative conformational energies (Δ*E*) in water and their RMSDs of the 45 local minima for the cationic Ac-dAba-NHMe at the CCSD(T), MP2, M06-2X, *ω*B97X-D, B2PLYP-D3BJ, and DSD-PBEP86-D3BJ levels of theory with various basis sets against the benchmark energies obtained by the sum of CCSD(T)/CBS-limit energies and solvation free energies are listed in Table 2. The Δ*E* value spanned up to 4.77 kcal/mol (conformer m45) at the CCSD(T)/CBS-limit//SMD M06-2X/6-31+G(d) level of theory in water. The RMSDs of Δ*E* values for the 45 local minima for the cationic Ac-dAba-NHMe in water at various levels of theory are depicted in Figure 3. In particular, the conformer m01 with a bifurcated H-bond (i.e., having *C*_7a_ and *C*_7b_ H-bonds) of the H–N^+^ group of the side chain with two C=O groups of the backbone was identified as the lowest-energy structure in water at all the levels of theory considered in this work.

**Table 2:** Relative energies (kcal/mol) of the 45 local minima of the cationic Ac-dAba-NHMe in water.^a^

Conf.	CCSD(T)		MP2					M06-2X			*ω*B97X-D			B2PLYP-D3BJ			DSD-PBEP86-D3BJ		
	CBS	DZ	DZ	aDZ	aTZ	aQZ	CBS	TZ	dTZ	dQZ	TZ	dTZ	dQZ	TZ	dTZ	dQZ	TZ	dTZ	dQZ
m01	0.00	0.00	0.00	0.00	0.00	0.00	0.00	0.00	0.00	0.00	0.00	0.00	0.00	0.00	0.00	0.00	0.00	0.00	0.00
m02	0.48	1.21	1.18	0.39	0.45	0.45	0.45	1.35	1.15	1.09	1.23	0.96	0.86	0.80	0.49	0.49	0.77	0.47	0.52
m03	0.75	1.93	1.56	0.04	0.28	0.34	0.38	1.29	0.93	1.00	1.90	1.43	1.41	1.56	0.92	1.08	1.24	0.54	0.82
m04	0.85	1.68	1.73	1.01	0.94	0.91	0.90	1.66	1.50	1.41	1.59	1.37	1.28	1.23	0.98	0.94	1.22	0.99	0.95
m05	0.87	1.30	1.26	0.40	0.64	0.75	0.83	1.26	1.10	1.14	1.12	0.87	0.90	1.06	0.73	0.86	1.03	0.68	0.88
m06	0.90	1.67	1.17	0.02	0.23	0.32	0.39	1.82	1.37	1.53	2.07	1.52	1.54	1.61	0.93	1.13	1.31	0.57	0.90
m07	1.05	2.19	1.90	0.57	0.68	0.72	0.76	1.24	0.90	1.04	1.94	1.51	1.59	1.81	1.26	1.42	1.50	0.88	1.15
m08	1.15	1.92	1.67	0.79	0.91	0.90	0.90	1.38	1.17	1.19	1.81	1.56	1.49	1.63	1.33	1.36	1.40	1.09	1.18
m09	1.33	1.77	1.68	1.21	1.16	1.21	1.25	1.57	1.33	1.44	1.52	1.25	1.31	1.58	1.25	1.37	1.49	1.14	1.32
m10	1.39	2.06	1.83	1.00	1.06	1.12	1.16	1.62	1.40	1.47	1.83	1.57	1.57	1.74	1.42	1.51	1.55	1.21	1.36
m11	1.54	3.17	2.87	1.04	1.20	1.22	1.24	1.87	1.41	1.50	2.62	2.06	2.05	2.48	1.77	1.90	2.12	1.34	1.61
m12	1.55	2.78	2.91	1.81	1.72	1.70	1.68	2.40	2.12	2.01	2.11	1.78	1.66	2.01	1.65	1.54	2.02	1.68	1.58
m13	1.72	2.06	2.00	1.35	1.58	1.63	1.66	1.77	1.72	1.72	1.56	1.45	1.40	1.73	1.59	1.63	1.73	1.56	1.67
m14	1.76	2.34	2.00	0.39	1.06	1.27	1.42	2.26	2.00	2.16	3.11	2.71	2.73	2.56	2.00	2.23	2.21	1.59	1.94
m15	1.85	1.02	0.77	0.45	1.09	1.39	1.60	1.18	1.34	1.48	1.47	1.65	1.76	1.54	1.66	1.85	1.54	1.60	1.82
m16	1.87	2.50	2.71	2.04	2.09	2.08	2.08	1.28	1.04	1.17	3.11	2.74	2.83	2.98	2.53	2.65	2.55	2.07	2.27
m17	1.89	2.81	2.59	1.33	1.61	1.65	1.68	2.97	2.62	2.59	3.18	2.67	2.58	2.66	2.06	2.12	2.42	1.80	1.98
m18	1.93	3.23	2.74	0.84	1.10	1.30	1.44	2.66	2.27	2.37	2.86	2.37	2.40	2.62	2.03	2.20	2.32	1.72	1.95
m19	1.96	2.14	1.93	1.61	1.70	1.73	1.75	2.04	1.89	1.98	2.35	2.13	2.15	2.20	1.96	2.06	2.02	1.76	1.93
m20	1.99	2.64	2.52	1.73	1.88	1.87	1.87	2.28	2.06	2.05	2.47	2.21	2.14	2.37	2.06	2.06	2.25	1.93	1.99
m21	2.00	1.89	1.91	1.30	1.74	1.90	2.02	2.20	2.18	2.21	2.08	1.96	1.97	2.06	1.85	2.00	2.04	1.79	2.01
m22	2.02	2.32	2.24	1.20	1.62	1.81	1.94	2.11	2.03	2.16	2.09	1.92	2.05	2.03	1.77	1.99	2.02	1.72	2.03
m23	2.03	2.19	2.12	1.88	1.88	1.92	1.96	1.78	1.65	1.82	2.10	1.93	2.03	2.22	2.00	2.18	2.07	1.80	2.06
m24	2.05	2.91	2.72	1.67	1.79	1.83	1.86	2.69	2.35	2.41	3.01	2.56	2.56	2.73	2.21	2.30	2.52	1.97	2.15
m25	2.09	2.72	2.42	1.44	1.71	1.76	1.79	3.26	2.88	2.90	3.25	2.75	2.67	2.76	2.15	2.26	2.54	1.90	2.14
m26	2.29	3.13	3.19	2.56	2.46	2.40	2.35	2.98	2.77	2.70	2.82	2.56	2.46	2.68	2.41	2.34	2.66	2.41	2.36
m27	2.51	3.50	3.22	1.67	2.05	2.15	2.23	2.95	2.55	2.83	3.52	3.00	3.15	3.24	2.57	2.90	2.92	2.17	2.66
m28	2.57	3.72	3.29	1.77	2.02	2.09	2.14	3.07	2.73	2.79	3.47	3.06	2.96	3.28	2.77	2.86	2.94	2.39	2.61
m29	2.59	4.29	3.93	1.63	1.92	2.10	2.23	2.91	2.48	2.62	3.39	2.85	2.89	3.38	2.70	2.87	3.05	2.31	2.60
m30	2.81	3.41	3.27	1.68	2.33	2.53	2.67	3.39	3.18	3.22	4.18	3.79	3.75	3.56	3.05	3.18	3.33	2.76	3.02
m31	2.87	2.59	2.51	2.20	2.56	2.69	2.79	2.59	2.62	2.73	3.50	3.48	3.57	3.08	3.01	3.16	2.93	2.82	3.02
m32	3.05	3.44	3.83	3.32	3.51	3.47	3.44	3.04	2.83	2.89	3.71	3.42	3.44	3.69	3.38	3.39	3.56	3.24	3.30
m33	3.06	4.15	4.08	2.76	2.97	2.98	2.99	3.96	3.68	3.62	4.34	3.89	3.79	3.86	3.33	3.32	3.68	3.13	3.22
m34	3.13	3.67	3.32	2.46	2.72	2.76	2.78	3.65	3.37	3.53	4.02	3.66	3.70	3.69	3.23	3.42	3.41	2.91	3.20
m35	3.28	4.20	3.93	2.45	2.72	2.89	3.01	4.23	3.95	3.91	4.22	3.82	3.78	3.88	3.42	3.50	3.69	3.22	3.36
m36	3.29	4.61	4.39	2.68	3.01	3.04	3.07	3.71	3.23	3.43	4.25	3.68	3.74	4.19	3.48	3.70	3.85	3.07	3.44
m37	3.49	4.53	4.21	2.76	3.08	3.13	3.17	4.14	3.90	3.75	4.42	4.09	3.84	4.15	3.74	3.64	3.96	3.52	3.53
m38	3.51	4.52	4.25	2.61	2.88	3.09	3.24	4.30	4.04	3.95	4.30	3.93	3.84	4.08	3.63	3.65	3.92	3.48	3.56
m39	3.52	4.26	4.29	2.92	3.39	3.48	3.54	4.01	3.73	3.84	4.45	4.00	4.05	4.22	3.65	3.84	4.00	3.37	3.70
m40	3.56	3.94	3.65	2.46	2.98	3.15	3.27	3.92	3.63	3.87	4.62	4.21	4.31	4.25	3.69	4.01	3.90	3.26	3.72
m41	3.67	4.12	3.94	2.44	3.18	3.36	3.49	3.86	3.61	3.86	4.42	3.96	4.13	4.29	3.67	4.05	3.99	3.29	3.83
m42	3.78	4.91	4.52	2.75	3.10	3.26	3.38	4.32	3.94	4.04	4.44	3.95	3.93	4.42	3.84	3.97	4.12	3.51	3.75
m43	3.90	3.61	3.00	1.54	2.56	2.98	3.29	3.13	3.12	3.33	4.87	4.73	4.88	4.52	4.28	4.56	3.97	3.65	4.03
m44	4.12	5.00	4.83	3.41	3.71	3.85	3.94	4.63	4.29	4.43	5.11	4.60	4.63	4.97	4.35	4.50	4.68	4.01	4.28
m45	4.77	4.89	4.82	4.21	4.37	4.56	4.70	4.75	4.60	4.69	5.04	4.83	4.84	5.03	4.78	4.91	4.84	4.59	4.77
RMSD^b^		0.85	0.71	0.79	0.46	0.34	0.27	0.58	0.39	0.38	0.85	0.50	0.51	0.61	0.19	0.29	0.39	0.19	0.12

a All structures were optimized at the SMD M06-2X/6-31+G(d) level of theory in water. Each relative energy was calculated by the sum of the single-point energy (Δ*E*_sp_) and the solvation free energy (Δ*G*_s_) as in Table 1. The CBS limit, cc-pVDZ, cc-pVTZ, aug-cc-pVDZ, aug-cc-pVTZ, aug-cc-pVQZ, def2-TZVP, and def2-QZVP basis sets are abbreviated as CBS, DZ, TZ, aDZ, aTZ, aQZ, dTZ, and dQZ, respectively.

b RMSD against the values obtained at the CCSD(T)/CBS limit level of theory.

The double-hybrid DSD-PBEP86-D3BJ/dQZ level of theory exhibited the best performance (RMSD = 0.12 kcal/mol) against the benchmark CCSD(T)/CBS-limit energies in water and was followed by the several levels of theory with small RMSD values in the order DSD-PBEP86-D3BJ/dTZ (0.19) = B2PLYP-D3BJ/dTZ (0.19) > MP2/CBS-limit (0.27) ≈ B2PLYP-D3BJ/dQZ (0.29), where RMSD values are in parentheses. In particular, the DSD-PBEP86-D3BJ/dQZ level of theory well predicted the order of conformational stability at the CCSD(T)/CBS-limit level of theory in water, except for the relative stabilities of conformer m04, m14, and m16 with marginal deviations.

The M06-2X/dQZ and M06-2X/dTZ levels of theory exhibited a better performance (RMSD = 0.38 and 0.39 kcal/mol, respectively) than the MP2/aTZ level of theory (RMSD = 0.46 kcal/mol) and the B2PLYP-D3BJ/TZ level of theory (RMSD = 0.61 kcal/mol), which are similar to those of the MP2/aQZ and DSD-PBEP86-D3BJ/TZ levels of theory (RMSD = 0.34 and 0.39 kcal/mol, respectively). However, the CCSD(T)/DZ, MP2/aDZ, and MP2/DZ levels of theory exhibited a little worse performance (RMSD = 0.85, 0.79, and 0.71 kcal/mol, respectively). The M06-2X/TZ, *ω*B97X-D/dQZ, *ω*B97X-D/dTZ, and *ω*B97X-D/TZ levels of theory exhibited the performance with RMSD = 0.58, 0.51, 0.50, and 0.85 kcal/mol, respectively.

Hence, the double-hybrid DSD-PBEP86-D3BJ/def2-QZVP level of theory is expected to provide the accurate relative conformational energies of the peptide with cationic side chains with RMSD ≈0.1 kcal/mol in water comparable to the benchmark CCSD(T)/CBS-limit energies. In particular, the M06-2X/dQZ level of theory exhibited a good performance (RMSD <0.4 kcal/mol) against the benchmark energies and may be an alternative level of theory with marginal deviations for the calculation of conformational energies of the larger cationic peptides in water.

### 3.3 Conformational free energies in water

The relative Gibbs free energies (Δ*G*) and populations of the 45 local minima for the cationic Ac-dAba-NHMe in water using single-point energies at the CCSD(T)/CBS-limit, DSD-PBEP86-D3BJ (with dQZ and dTZ basis sets), and M06-2X/dQZ levels of theory are listed in Table 3. Conformer m02 with a *H*_14_–helix backbone was identified as the most preferred conformation in water at all the CCSD(T)/CBS-limit, DSD-PBEP86-D3BJ/dQZ, DSD-PBEP86-D3BJ/dTZ, and M06-2X/dQZ levels of theory (populated at 40, 41, 34, and 25%, respectively). The second most preferred conformer m03 also adopted a *H*_14_–helix backbone at the same levels of theory (populated at 17, 16, 19, and 19%, respectively). However, the third most preferred conformation was m06 with a *H*_14_–helix backbone at the first three levels of theory (populated at 7, 8, and 10%, respectively), whereas the corresponding conformation was m23 with *C*_6_/*C*_7b_ H-bonds with a population of 9% and conformer m06 was populated at 4% at the M06-2X/dQZ level of theory. Hence, the *H*_14_–structure appeared to be the most feasible conformation for the cationic Ac-dAba-NHMe in water populated at 48–64% at all the levels of theory considered in this work.

**Table 3:** Free energies (kcal/mol) and populations (%) of the 45 local minima of the cationic Ac-dAba-NHMe in water.^a^

Conf.	CCSD(T)		DSD-PBEP86-D3BJ				M06-2X	
	CBS limit		def2-QZVP		def2-TZVP		def2-QZVP	
	Δ*G*	*w*	Δ*G*	*w*	Δ*G*	*w*	Δ*G*	*w*
m02	0.00	40.0	0.00	40.7	0.00	33.8	0.00	24.9
m03	0.52	16.5	0.57	15.7	0.34	19.1	0.18	18.5
m06	1.03	7.1	1.00	7.6	0.72	10.0	1.05	4.2
m09	1.41	3.7	1.37	4.1	1.24	4.2	0.91	5.3
m23	1.45	3.4	1.45	3.5	1.25	4.1	0.64	8.5
m12	1.46	3.4	1.45	3.5	1.60	2.3	1.31	2.7
m05	1.48	3.3	1.46	3.5	1.31	3.7	1.14	3.6
m07	1.48	3.3	1.54	3.0	1.33	3.6	0.87	5.8
m01	1.55	2.9	1.51	3.2	1.57	2.4	0.94	5.1
m04	1.64	2.5	1.70	2.3	1.79	1.7	1.59	1.7
m11	1.64	2.5	1.68	2.4	1.46	2.9	0.99	4.7
m14	1.72	2.2	1.87	1.7	1.57	2.4	1.52	1.9
m19	1.93	1.6	1.87	1.7	1.75	1.8	1.35	2.5
m17	2.21	1.0	2.26	0.9	2.13	0.9	2.30	0.5
m08	2.36	0.7	2.35	0.8	2.31	0.7	1.79	1.2
m27	2.41	0.7	2.53	0.6	2.09	1.0	2.13	0.7
m28	2.51	0.6	2.51	0.6	2.34	0.6	2.12	0.7
m29	2.65	0.5	2.63	0.5	2.39	0.6	2.08	0.7
m26	2.71	0.4	2.74	0.4	2.85	0.3	2.52	0.4
m10	2.72	0.4	2.66	0.5	2.56	0.4	2.20	0.6
m16	2.77	0.4	3.13	0.2	2.99	0.2	1.46	2.1
m22	2.89	0.3	2.86	0.3	2.61	0.4	2.43	0.4
m21	2.93	0.3	2.90	0.3	2.74	0.3	2.53	0.3
m24	2.97	0.3	3.04	0.2	2.90	0.3	2.73	0.3
m15	2.98	0.3	2.92	0.3	2.75	0.3	2.01	0.8
m25	3.00	0.3	3.01	0.3	2.83	0.3	3.20	0.1
m18	3.04	0.2	3.02	0.2	2.84	0.3	2.87	0.2
m38	3.08	0.2	3.10	0.2	3.07	0.2	2.92	0.2
m40	3.09	0.2	3.22	0.2	2.81	0.3	2.80	0.2
m30	3.21	0.2	3.38	0.1	3.18	0.2	3.01	0.2
m36	3.39	0.1	3.50	0.1	3.18	0.2	2.93	0.2
m32	3.62	0.1	3.83	0.1	3.82	0.1	2.85	0.2
m13	3.69	0.1	3.60	0.1	3.54	0.1	3.08	0.1
m39	3.75	0.1	3.90	0.1	3.62	0.1	3.47	0.1
m20	3.82	0.1	3.78	0.1	3.78	0.1	3.28	0.1
m33	4.09	0.0	4.21	0.0	4.18	0.0	4.04	0.0
m31	4.11	0.0	4.22	0.0	4.08	0.0	3.36	0.1
m41	4.14	0.0	4.26	0.0	3.78	0.1	3.72	0.0
m34	4.23	0.0	4.27	0.0	4.03	0.0	4.03	0.0
m37	4.29	0.0	4.29	0.0	4.33	0.0	3.94	0.0
m35	4.37	0.0	4.41	0.0	4.32	0.0	4.39	0.0
m44	4.38	0.0	4.51	0.0	4.29	0.0	4.09	0.0
m42	4.60	0.0	4.54	0.0	4.35	0.0	4.26	0.0
m43	5.99	0.0	6.09	0.0	5.75	0.0	4.82	0.0
m45	6.73	0.0	6.70	0.0	6.56	0.0	6.04	0.0

a All structures were optimized at the SMD M06-2X/6-31+G(d) level of theory in water. Each relative Gibbs free energy (Δ*G*) was calculated by the sum of Δ*E* (Table 2) and the enthalpic and entropic contributions (i.e., Δ*H*_c_ and −*T*Δ*S*_c_ in Table S1 of the Supplementary material).

As described in earlier section, conformer m01 was the lowest-energy structure at all the levels of theory. However, its free energy was 1.55, 1.51, 1.57, and 0.94 kcal/mol at the CCSD(T)/CBS-limit, DSD-PBEP86-D3BJ/dQZ, DSD-PBEP86-D3BJ/dTZ, and M06-2X/dQZ levels of theory, respectively. The increased Δ*G* value for m01 was ascribed to the decrease of the entropic contribution (see Table S1 of the Supplementary material), i.e., the less conformational flexibility due to the formation of a bifurcated H-bond with *C*_7a_ and *C*_7b_ H-bonds (see Figure 2).

**Figure 4:**
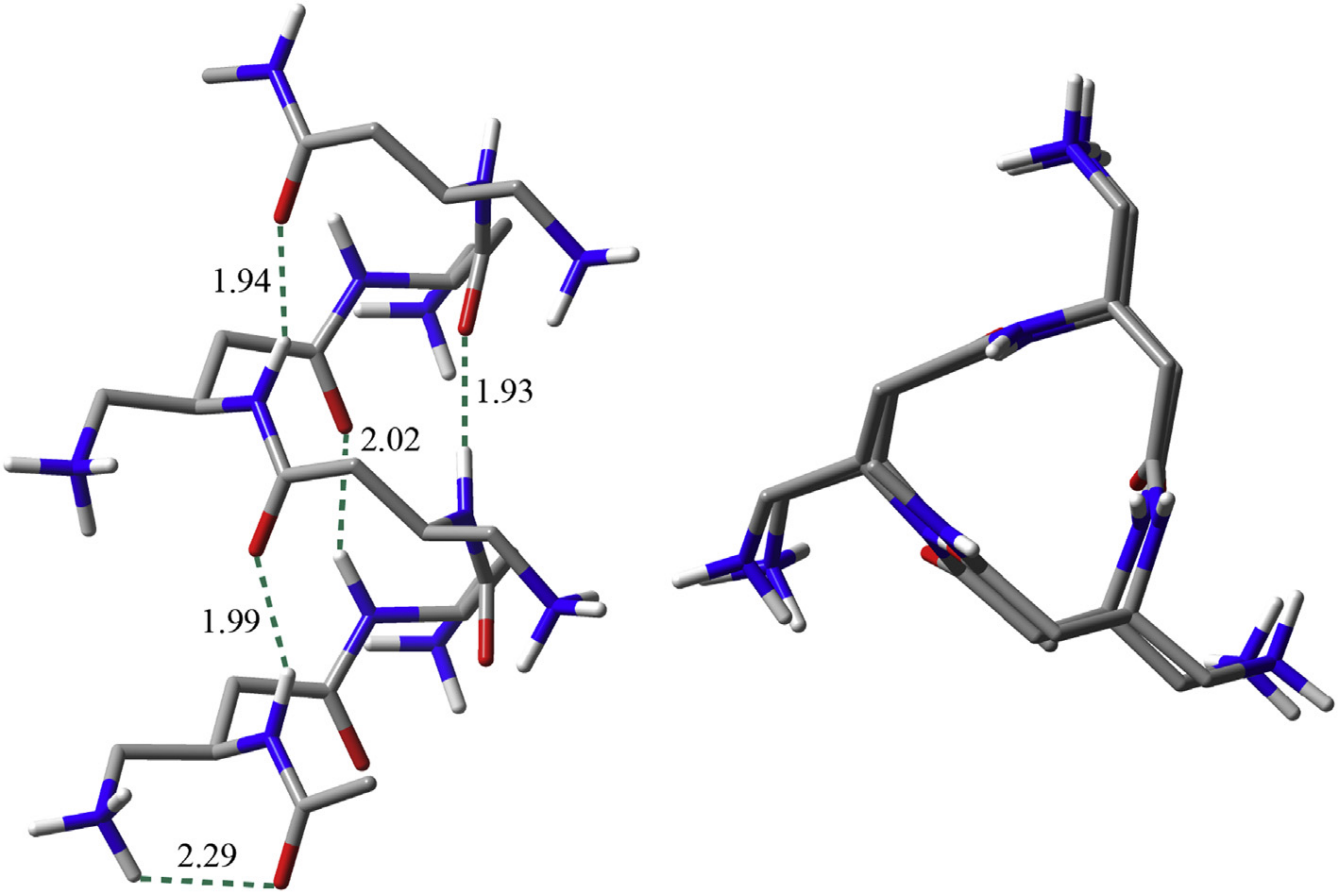
Structure of the hexamer from conformer m03 of the cationic Ac-dAba-NHMe in water optimized at the SMD M06-2X/6-31G(d) level of theory: (left) view along the helix axis and (right) view perpendicular to the helical axis. H-bonds are represented by dotted lines and their distances are in Å.

From the two most preferred conformers m02 and m03 in water, two hexamers of the cationic Ac-dAba-NHMe were built and optimized at the SMD M06-2X/6-31G(d) level of theory in water. Two optimized structures were quite similar to each other but the hexamer from conformer m03 was 0.52 kcal/mol more stable than that from conformer m02. The optimized structure of the hexamer from conformer 03 is depicted in Figure 4 and its Cartesian coordinates is listed in Table S7 of the Supplementary material. The hexamer adopted a left-handed 3_14_-helix with radius = 2.57 Å, residues/turn = 3.0, and rise/residue = 1.71 Å. This 3_14_-helix has a slightly narrower radius and a longer rise than the regular 3_14_-helix of β-peptides with radius = 2.7 Å and rise/residue = 1.56 Å [45]. The mean backbone torsion angles for the 3_14_-helix of the hexamer from conformer m03 are *φ* = −132°, *θ* = 58°, and *ψ* = −139°, whereas those are *φ* = −150°, *θ* = 62°, and *ψ* = −138° for the *H*_14_-helix of the tetramer of βAbu optimized at the HF/6-31G(d) level of theory [43]. We monitored the H-bond distances *d*(C=O···H–N) for the 3_14_-helix of the hexamer of conformer m03, whose mean value is 1.97 Å for backbone and the distance for the first *C*_7a_ H-bond is 2.29 Å (see Figure 4). Hence, the 3_14_-helices of oligomers or polymers of the cationic dAba residues are expected to be the active conformation to exhibit the ability to bridge between charged lipid head groups that might cause a local depression or invagination of the membrane of fungi, suggested from the membrane leakage experiments [26].

## 4 Conclusions

We explored the conformational preferences of the cationic nylon-3 βNM (dAba) dipeptide in water as the first step to understand the mode of action of polymers of βNM against phylogenetically diverse and intrinsically drug-resistant pathogenic fungi. At the SMD M06-2X/6-31+G(d) level of theory in water, the 45 local minima of the cationic Ac-dAba-NHMe with Δ*E* < 5 kcal/mol were identified. The CCSD(T), MP2, M06-2X, *ω*B97X-D, B2PLYP-D3BJ, and DSD-PBEP86-D3BJ levels of theory with various basis sets were assessed for relative energies of these 45 local minima against the benchmark CCSD(T)/CBS-limit energies in water.

The double-hybrid DSD-PBEP86-D3BJ/def2-QZVP level of theory exhibited the best performance (RMSD = 0.12 kcal/mol) in water against to the benchmark CCSD(T)/CBS-limit energies and was followed by the several levels of theory such as DSD-PBEP86-D3BJ/def2-TZVP, B2PLYP-D3BJ/def2-TZVP, MP2/CBS-limit, and B2PLYP-D3BJ/def2-QZVP with small RMSD values (0.19–0.29 kcal/mol). In particular, the DSD-PBEP86-D3BJ/def2-QZVP level of theory well predicted the order of conformational stability at the CCSD(T)/CBS-limit level of theory in water. The M06-2X/dQZ and M06-2X/dTZ levels of theory exhibited a better performance (RMSD = 0.38 and 0.39 kcal/mol, respectively) than the MP2/aTZ and B2PLYP-D3BJ/TZ levels of theory. Hence, the M06-2X/def2-QZVP level of theory may be an alternative level of theory with marginal deviations to reduce the computational times without any significant loss in accuracy for conformational energies of relatively longer cationic peptides in water.

In particular, the *H*_14_–helical structures appeared to be the most feasible conformations for the cationic Ac-dAba-NHMe populated at 48–64% by relative free energies in water. The hexamer built from the *H*_14_–structure of the cationic Ac-dAba-NHMe adopted a left-handed 3_14_-helix, which has a slightly narrower radius and a longer rise than the regular 3_14_-helix of β-peptides. Hence, the 3_14_-helices of oligomers or polymers of the cationic dAba residues are expected to be the active conformation to exhibit the ability to bridge between charged lipid head groups that might cause a local depression or invagination of the membrane of fungi.

## Declarations

### Author contribution statement

Young Kee Kang: Conceived and designed the analysis; Analyzed and interpreted the data; Contributed analysis tools or data; Wrote the paper.

Hae Sook Park: Analyzed and interpreted the data; Contributed analysis tools or data; Wrote the paper.

### Funding statement

This work was supported by Basic Science Research Program through the 10.13039/501100003725National Research Foundation of Korea (NRF) funded by the Ministry of Education (2018R1D1A3B07043412).

### Competing interest statement

The authors declare no conflict of interest.

### Additional information

No additional information is available for this paper.

## Appendix A Supplementary data

Supplementary_Material-R3 Supplementary_Material-R3
