# Supplementary material for: Conformational preferences of cationic β-peptide in water studied by CCSD(T), MP2, and DFT methods
Source: Heliyon. 2020 Aug 14;6(8):e04721. doi: 10.1016/j.heliyon.2020.e04721 (PMC7452530; doi:10.1016/j.heliyon.2020.e04721)
Supplement: Supplementary_Material-R3 [file mmc1.doc]

**Supplementary Material**

**Conformational preferences of cationic β-peptide in water studied by CCSD(T), MP2, and DFT methods**

###### Young Kee Kang^a^,*, Hae Sook Park^b^

^a^ *Department of Chemistry, Chungbuk National University, Cheongju, Chungbuk 28644, Republic of Korea. E-mail: ykkang@chungbuk.ac.kr*

^b^ *Department of Nursing, Cheju Halla University, Cheju 63092, Republic of Korea*

**Page Contents**

s1 Table of Contents

s2 **Table S1** Absolute and relative thermodynamic properties and solvation free energies of the 45 local minima of the cationic Ac-dAba-NHMe optimized at the SMD M06-2X/6-31+G(d) level of theory in water

s3−s4 **Table S2** Absolute and relative electronic energies by M06-2X and *ω*B97X-D functionals of the 45 local minima of the cationic Ac-dAba-NHMe in water

s5−s6 **Table S3** Absolute and relative electronic energies by double-hybrid B2PLYP-D3BJ and DSD-PBEP86-D3BJ functionals of the 45 local minima of the cationic Ac-dAba-NHMe in water

s7−s8 **Table S4** Absolute and relative electronic energies by MP2 and CCSD(T) methods of the 45 local minima of the cationic Ac-dAba-NHMe in water

s9 **Table S5** Absolute and relative electronic energies by MP2/CBS limit and CCSD(T)/CBS limit methods of the 45 local minima of the cationic Ac-dAba-NHMe in water

s10−s24 **Table S6** Cartesian coordinates of the 45 local minima of the cationic Ac-dAba-NHMe optimized at the SMD M06-2X/6-31+G(d) level of theory in water

s24 −s25 **Table S7** Cartesian coordinates of the *H*_14_-helical structure of hexamer built from conformer m03 optimized at the SMD M06-2X/6-31G(d) level of theory in water

| **Table S1**  Absolute and relative thermodynamic properties and solvation free energies of the 45 local minima of the cationic Ac-dAba-NHMe with Δ*E* < 5 kcal/mol optimized at the SMD M06-2X/6-31+G(d) level of theory in water. | | | | | | | |
| --- | --- | --- | --- | --- | --- | --- | --- |
| Conf. | *E*_e_^a^ | *H*^a^ | *G*^a^ | Δ*H*_c_^b^ | −*T*Δ*S*_c_^c^ | *E*_e,s1_^d^ | Δ*G*_s_^e^ |
| m01 | -590.769341 | -590.516901 | -590.572625 | 0.00 | 0.95 | -590.664859 | -65.56 |
| m02 | -590.767210 | -590.514706 | -590.573730 | 0.04 | -1.13 | -590.641751 | -78.73 |
| m03 | -590.768025 | -590.515446 | -590.574131 | 0.09 | -0.91 | -590.634244 | -83.95 |
| m04 | -590.766613 | -590.514188 | -590.571116 | -0.01 | 0.19 | -590.647323 | -74.86 |
| m05 | -590.767600 | -590.515039 | -590.572377 | 0.08 | -0.07 | -590.646699 | -75.87 |
| m06 | -590.767645 | -590.516444 | -590.573185 | -0.78 | 0.31 | -590.625707 | -89.07 |
| m07 | -590.768334 | -590.515663 | -590.573403 | 0.14 | -0.32 | -590.644129 | -77.94 |
| m08 | -590.767403 | -590.514516 | -590.571224 | 0.28 | 0.33 | -590.643737 | -77.60 |
| m09 | -590.767527 | -590.515379 | -590.573150 | -0.18 | -0.34 | -590.652214 | -72.36 |
| m10 | -590.766991 | -590.514147 | -590.570624 | 0.25 | 0.47 | -590.641399 | -78.81 |
| m11 | -590.767178 | -590.514830 | -590.572772 | -0.06 | -0.45 | -590.636957 | -81.71 |
| m12 | -590.765613 | -590.513216 | -590.571512 | -0.03 | -0.67 | -590.645430 | -75.42 |
| m13 | -590.766258 | -590.513223 | -590.568877 | 0.37 | 0.99 | -590.644692 | -76.28 |
| m14 | -590.766570 | -590.514275 | -590.572379 | -0.09 | -0.55 | -590.619546 | -92.26 |
| m15 | -590.767690 | -590.515585 | -590.571634 | -0.21 | 0.74 | -590.656977 | -69.47 |
| m16 | -590.767580 | -590.515284 | -590.571899 | -0.09 | 0.39 | -590.660141 | -67.42 |
| m17 | -590.764936 | -590.511993 | -590.570188 | 0.32 | -0.61 | -590.618524 | -91.88 |
| m18 | -590.766016 | -590.514596 | -590.570004 | -0.64 | 1.14 | -590.617098 | -93.45 |
| m19 | -590.766146 | -590.513843 | -590.571942 | -0.09 | -0.55 | -590.643012 | -77.27 |
| m20 | -590.765855 | -590.512803 | -590.568692 | 0.38 | 0.84 | -590.637483 | -80.55 |
| m21 | -590.765747 | -590.514332 | -590.570018 | -0.64 | 0.97 | -590.641306 | -78.09 |
| m22 | -590.765990 | -590.513522 | -590.570362 | 0.02 | 0.24 | -590.643770 | -76.69 |
| m23 | -590.767134 | -590.515003 | -590.573798 | -0.19 | -0.98 | -590.651961 | -72.27 |
| m24 | -590.765590 | -590.512787 | -590.569873 | 0.23 | 0.09 | -590.624359 | -88.62 |
| m25 | -590.764697 | -590.511624 | -590.569003 | 0.40 | -0.09 | -590.615355 | -93.71 |
| m26 | -590.764494 | -590.511987 | -590.569576 | 0.04 | -0.23 | -590.645655 | -74.57 |
| m27 | -590.765635 | -590.513170 | -590.571542 | 0.02 | -0.72 | -590.631117 | -84.41 |
| m28 | -590.764938 | -590.512284 | -590.570789 | 0.13 | -0.80 | -590.617554 | -92.48 |
| m29 | -590.765604 | -590.513133 | -590.571252 | 0.02 | -0.56 | -590.626631 | -87.21 |
| m30 | -590.764096 | -590.511518 | -590.569212 | 0.09 | -0.29 | -590.609676 | -96.90 |
| m31 | -590.765114 | -590.512735 | -590.568896 | -0.04 | 0.67 | -590.645841 | -74.85 |
| m32 | -590.764813 | -590.512555 | -590.569662 | -0.11 | 0.08 | -590.650277 | -71.87 |
| m33 | -590.763241 | -590.510167 | -590.567355 | 0.40 | 0.03 | -590.620926 | -89.30 |
| m34 | -590.764095 | -590.511191 | -590.568089 | 0.29 | 0.21 | -590.628608 | -85.02 |
| m35 | -590.762910 | -590.511443 | -590.566932 | -0.61 | 1.09 | -590.611978 | -94.71 |
| m36 | -590.764391 | -590.511788 | -590.569986 | 0.10 | -0.61 | -590.629082 | -84.91 |
| m37 | -590.762438 | -590.509374 | -590.566924 | 0.39 | -0.20 | -590.602958 | -100.08 |
| m38 | -590.762727 | -590.510141 | -590.569161 | 0.09 | -1.12 | -590.608673 | -96.67 |
| m39 | -590.763212 | -590.510316 | -590.568588 | 0.29 | -0.65 | -590.624419 | -87.09 |
| m40 | -590.763845 | -590.511621 | -590.570338 | -0.14 | -0.93 | -590.621787 | -89.14 |
| m41 | -590.763466 | -590.510594 | -590.568475 | 0.27 | -0.41 | -590.623415 | -87.88 |
| m42 | -590.763204 | -590.510289 | -590.567644 | 0.30 | -0.08 | -590.613315 | -94.06 |
| m43 | -590.764860 | -590.512078 | -590.567285 | 0.21 | 1.27 | -590.632122 | -83.29 |
| m44 | -590.762426 | -590.509697 | -590.567767 | 0.18 | -0.53 | -590.616899 | -91.32 |
| m45 | -590.761946 | -590.509008 | -590.564573 | 0.31 | 1.04 | -590.632512 | -81.22 |
| ^a^ *E*_e_, *H*, and *G* are absolute electronic energy, enthalpy, and Gibbs free energy of each conformer calculated at the SMD M06-2X/6-31+G(d) level of theory in water, respectively. Units in hartrees. ^b^ The enthalpic contribution (kcal/mol) calculated by *H* – *E*_e_. ^c^ The entropic contribution (kcal/mol) calculated by *G* – *H*. ^d^ The single-point energy (hartrees) calculated at the M06-2X/6-31+G(d) level of theory. ^e^ The solvation free energy (kcal/mol) calculated by *E*_e_ – *E*_e,s1_. | | | | | | | |

| **Table S2**  Absolute and relative electronic energies by M06-2X and *ω*B97X-D functionals of the 45 local minima of the cationic Ac-dAba-NHMe optimized at the SMD M06-2X/6-31+G(d) level of theory in water.^a^ | | | | | | | | | | | | |
| --- | --- | --- | --- | --- | --- | --- | --- | --- | --- | --- | --- | --- |
| Conf. | M06-2X | | | | | | *ω*B97X-D | | | | | |
|  | cc-pVTZ | | def2-TZVP | | def2-QZVP | | cc-pVTZ | | def2-TZVP | | def2-QZVP | |
|  | *E*_e_ | Δ*E* | *E*_e_ | Δ*E* | *E*_e_ | Δ*E* | *E*_e_ | Δ*E* | *E*_e_ | Δ*E* | *E*_e_ | Δ*E* |
| m01 | -590.886319 | 0.00 | -590.891470 | 0.00 | -590.945968 | 0.00 | -590.958075 | 0.00 | -590.972739 | 0.00 | -591.014827 | 0.00 |
| m02 | -590.863193 | 14.51 | -590.868665 | 14.31 | -590.923260 | 14.25 | -590.935144 | 14.39 | -590.950237 | 14.12 | -590.992475 | 14.03 |
| m03 | -590.854967 | 19.67 | -590.860691 | 19.31 | -590.915070 | 19.39 | -590.925741 | 20.29 | -590.941161 | 19.82 | -590.983276 | 19.80 |
| m04 | -590.868864 | 10.95 | -590.874275 | 10.79 | -590.928908 | 10.71 | -590.940734 | 10.88 | -590.955746 | 10.66 | -590.997977 | 10.57 |
| m05 | -590.867897 | 11.56 | -590.873292 | 11.41 | -590.927737 | 11.44 | -590.939871 | 11.42 | -590.954932 | 11.17 | -590.996970 | 11.21 |
| m06 | -590.845961 | 25.33 | -590.851826 | 24.88 | -590.906080 | 25.03 | -590.917319 | 25.57 | -590.932859 | 25.02 | -590.974910 | 25.05 |
| m07 | -590.864628 | 13.61 | -590.870319 | 13.27 | -590.924586 | 13.42 | -590.935258 | 14.32 | -590.950605 | 13.89 | -590.992564 | 13.97 |
| m08 | -590.864931 | 13.42 | -590.870429 | 13.20 | -590.924892 | 13.23 | -590.936012 | 13.84 | -590.951071 | 13.60 | -590.993264 | 13.53 |
| m09 | -590.872984 | 8.37 | -590.878516 | 8.13 | -590.932845 | 8.23 | -590.944827 | 8.31 | -590.959917 | 8.05 | -591.001902 | 8.11 |
| m10 | -590.862633 | 14.86 | -590.868130 | 14.65 | -590.922515 | 14.72 | -590.934047 | 15.08 | -590.949133 | 14.81 | -590.991210 | 14.82 |
| m11 | -590.857607 | 18.02 | -590.863478 | 17.57 | -590.917842 | 17.65 | -590.928161 | 18.77 | -590.943715 | 18.21 | -590.985819 | 18.20 |
| m12 | -590.866793 | 12.25 | -590.872396 | 11.97 | -590.927062 | 11.86 | -590.939005 | 11.97 | -590.954206 | 11.63 | -590.996477 | 11.51 |
| m13 | -590.866412 | 12.49 | -590.871649 | 12.44 | -590.926141 | 12.44 | -590.938499 | 12.28 | -590.953340 | 12.17 | -590.995506 | 12.12 |
| m14 | -590.840170 | 28.96 | -590.845741 | 28.70 | -590.899979 | 28.86 | -590.910584 | 29.80 | -590.925878 | 29.41 | -590.967942 | 29.42 |
| m15 | -590.878205 | 5.09 | -590.883099 | 5.25 | -590.937386 | 5.39 | -590.949501 | 5.38 | -590.963886 | 5.56 | -591.005797 | 5.67 |
| m16 | -590.881321 | 3.14 | -590.886862 | 2.89 | -590.941148 | 3.02 | -590.950158 | 4.97 | -590.965413 | 4.60 | -591.007366 | 4.68 |
| m17 | -590.839660 | 29.28 | -590.845367 | 28.93 | -590.899907 | 28.90 | -590.911077 | 29.49 | -590.926550 | 28.98 | -590.968788 | 28.89 |
| m18 | -590.837639 | 30.55 | -590.843421 | 30.15 | -590.897756 | 30.25 | -590.909083 | 30.74 | -590.924526 | 30.25 | -590.966562 | 30.29 |
| m19 | -590.864423 | 13.74 | -590.869804 | 13.60 | -590.924155 | 13.69 | -590.935685 | 14.05 | -590.950693 | 13.83 | -590.992750 | 13.85 |
| m20 | -590.858804 | 17.27 | -590.864293 | 17.05 | -590.918807 | 17.04 | -590.930249 | 17.46 | -590.945329 | 17.20 | -590.987531 | 17.13 |
| m21 | -590.862852 | 14.73 | -590.868042 | 14.70 | -590.922491 | 14.73 | -590.934801 | 14.60 | -590.949649 | 14.49 | -590.991735 | 14.49 |
| m22 | -590.865212 | 13.25 | -590.870497 | 13.16 | -590.924781 | 13.30 | -590.937005 | 13.22 | -590.951936 | 13.05 | -590.993825 | 13.18 |
| m23 | -590.872794 | 8.49 | -590.878156 | 8.35 | -590.932384 | 8.52 | -590.944042 | 8.81 | -590.958969 | 8.64 | -591.000899 | 8.74 |
| m24 | -590.845288 | 25.75 | -590.850979 | 25.41 | -590.905380 | 25.47 | -590.916522 | 26.07 | -590.931907 | 25.62 | -590.973991 | 25.63 |
| m25 | -590.836266 | 31.41 | -590.842012 | 31.04 | -590.896489 | 31.05 | -590.908033 | 31.40 | -590.923500 | 30.90 | -590.965711 | 30.82 |
| m26 | -590.867212 | 11.99 | -590.872703 | 11.78 | -590.927307 | 11.71 | -590.939216 | 11.83 | -590.954296 | 11.57 | -590.996552 | 11.47 |
| m27 | -590.851577 | 21.80 | -590.857363 | 21.40 | -590.911414 | 21.68 | -590.922423 | 22.37 | -590.937930 | 21.84 | -590.979765 | 22.00 |
| m28 | -590.838526 | 29.99 | -590.844222 | 29.65 | -590.898625 | 29.71 | -590.909640 | 30.39 | -590.924957 | 29.98 | -590.967209 | 29.88 |
| m29 | -590.847192 | 24.55 | -590.853019 | 24.13 | -590.907310 | 24.26 | -590.918188 | 25.03 | -590.933703 | 24.50 | -590.975730 | 24.53 |
| m30 | -590.830982 | 34.72 | -590.836456 | 34.52 | -590.890906 | 34.55 | -590.901473 | 35.52 | -590.916755 | 35.13 | -590.958920 | 35.08 |
| m31 | -590.867395 | 11.88 | -590.872499 | 11.90 | -590.926823 | 12.01 | -590.937704 | 12.78 | -590.952400 | 12.76 | -590.994347 | 12.85 |
| m32 | -590.871421 | 9.35 | -590.876904 | 9.14 | -590.931309 | 9.20 | -590.942111 | 10.02 | -590.957235 | 9.73 | -590.999299 | 9.74 |
| m33 | -590.842174 | 27.70 | -590.847774 | 27.42 | -590.902366 | 27.36 | -590.913333 | 28.08 | -590.928704 | 27.63 | -590.970954 | 27.53 |
| m34 | -590.849501 | 23.10 | -590.855101 | 22.82 | -590.909342 | 22.98 | -590.920658 | 23.48 | -590.935897 | 23.12 | -590.977927 | 23.16 |
| m35 | -590.833135 | 33.37 | -590.838718 | 33.10 | -590.893286 | 33.06 | -590.904901 | 33.37 | -590.920204 | 32.97 | -590.962348 | 32.93 |
| m36 | -590.849584 | 23.05 | -590.855501 | 22.57 | -590.909670 | 22.78 | -590.920479 | 23.59 | -590.936051 | 23.02 | -590.978037 | 23.09 |
| m37 | -590.824727 | 38.65 | -590.830257 | 38.41 | -590.884999 | 38.26 | -590.896031 | 38.93 | -590.911217 | 38.61 | -590.953716 | 38.35 |
| m38 | -590.829902 | 35.40 | -590.835463 | 35.14 | -590.890102 | 35.06 | -590.901643 | 35.41 | -590.916907 | 35.04 | -590.959131 | 34.95 |
| m39 | -590.845615 | 25.54 | -590.851211 | 25.26 | -590.905537 | 25.37 | -590.916669 | 25.98 | -590.932056 | 25.53 | -590.974065 | 25.58 |
| m40 | -590.842493 | 27.50 | -590.848104 | 27.21 | -590.902226 | 27.45 | -590.913138 | 28.20 | -590.928453 | 27.79 | -590.970381 | 27.89 |
| m41 | -590.844595 | 26.18 | -590.850149 | 25.93 | -590.904246 | 26.18 | -590.915461 | 26.74 | -590.930865 | 26.28 | -590.972674 | 26.45 |
| m42 | -590.834023 | 32.82 | -590.839779 | 32.44 | -590.894118 | 32.54 | -590.905595 | 32.93 | -590.921031 | 32.45 | -590.963164 | 32.42 |
| m43 | -590.853073 | 20.86 | -590.858246 | 20.85 | -590.912399 | 21.07 | -590.922061 | 22.60 | -590.936942 | 22.46 | -590.978791 | 22.61 |
| m44 | -590.837897 | 30.39 | -590.843582 | 30.05 | -590.897860 | 30.19 | -590.908882 | 30.87 | -590.924361 | 30.36 | -590.966401 | 30.39 |
| m45 | -590.853803 | 20.40 | -590.859186 | 20.26 | -590.913547 | 20.34 | -590.925089 | 20.70 | -590.940095 | 20.48 | -590.982157 | 20.50 |
| ^a^ Units for *E*_e_ and Δ*E* in hartrees and kcal/mol, respectively. | | | | | | | | | | | | |

| **Table S3**  Absolute and relative electronic energies by double-hybrid B2PLYP-D3BJ and DSD-PBEP86-D3BJ functionals of the 45 local minima of the cationic Ac-dAba-NHMe optimized at the SMD M06-2X/6-31+G(d) level of theory in water.^a^ | | | | | | | | | | | | |
| --- | --- | --- | --- | --- | --- | --- | --- | --- | --- | --- | --- | --- |
| Conf. | B2PLYP-D3BJ | | | | | | DSD-PBEP86-D3BJ | | | | | |
|  | cc-pVTZ | | def2-TZVP | | def2-QZVP | | cc-pVTZ | | def2-TZVP | | def2-QZVP | |
|  | *E*_e_ | Δ*E* | *E*_e_ | Δ*E* | *E*_e_ | Δ*E* | *E*_e_ | Δ*E* | *E*_e_ | Δ*E* | *E*_e_ | Δ*E* |
| m01 | -590.679036 | 0.00 | -590.677436 | 0.00 | -590.773872 | 0.00 | -590.092048 | 0.00 | -590.078971 | 0.00 | -590.216989 | 0.00 |
| m02 | -590.656782 | 13.96 | -590.655678 | 13.65 | -590.752114 | 13.65 | -590.069842 | 13.93 | -590.057252 | 13.63 | -590.195186 | 13.68 |
| m03 | -590.647256 | 19.94 | -590.646665 | 19.31 | -590.742856 | 19.46 | -590.060767 | 19.63 | -590.048806 | 18.93 | -590.186376 | 19.21 |
| m04 | -590.662270 | 10.52 | -590.661073 | 10.27 | -590.757573 | 10.23 | -590.075295 | 10.51 | -590.062591 | 10.28 | -590.200660 | 10.25 |
| m05 | -590.660934 | 11.36 | -590.659849 | 11.04 | -590.756078 | 11.17 | -590.073980 | 11.34 | -590.061467 | 10.98 | -590.199162 | 11.19 |
| m06 | -590.639009 | 25.12 | -590.638493 | 24.44 | -590.734614 | 24.63 | -590.052508 | 24.81 | -590.040605 | 24.07 | -590.178097 | 24.40 |
| m07 | -590.656424 | 14.19 | -590.655707 | 13.63 | -590.751879 | 13.80 | -590.069928 | 13.88 | -590.057842 | 13.26 | -590.195432 | 13.53 |
| m08 | -590.657249 | 13.67 | -590.656138 | 13.36 | -590.752527 | 13.39 | -590.070630 | 13.44 | -590.058057 | 13.12 | -590.195931 | 13.21 |
| m09 | -590.665683 | 8.38 | -590.664618 | 8.04 | -590.760852 | 8.17 | -590.078835 | 8.29 | -590.066325 | 7.94 | -590.204052 | 8.12 |
| m10 | -590.655157 | 14.98 | -590.654067 | 14.66 | -590.750354 | 14.76 | -590.068463 | 14.80 | -590.055927 | 14.46 | -590.193704 | 14.61 |
| m11 | -590.649346 | 18.63 | -590.648881 | 17.92 | -590.745106 | 18.05 | -590.062933 | 18.27 | -590.051089 | 17.50 | -590.188681 | 17.76 |
| m12 | -590.660135 | 11.86 | -590.659106 | 11.50 | -590.755725 | 11.39 | -590.073132 | 11.87 | -590.060600 | 11.53 | -590.198767 | 11.43 |
| m13 | -590.659198 | 12.45 | -590.657815 | 12.31 | -590.754183 | 12.36 | -590.072211 | 12.45 | -590.059397 | 12.28 | -590.197250 | 12.39 |
| m14 | -590.632418 | 29.25 | -590.631701 | 28.70 | -590.727781 | 28.92 | -590.045984 | 28.91 | -590.033901 | 28.28 | -590.171353 | 28.64 |
| m15 | -590.670350 | 5.45 | -590.668564 | 5.57 | -590.764699 | 5.76 | -590.083369 | 5.45 | -590.070186 | 5.51 | -590.207862 | 5.73 |
| m16 | -590.671337 | 4.83 | -590.670446 | 4.39 | -590.766688 | 4.51 | -590.085020 | 4.41 | -590.072712 | 3.93 | -590.210418 | 4.12 |
| m17 | -590.632862 | 28.97 | -590.632221 | 28.37 | -590.728569 | 28.43 | -590.046254 | 28.74 | -590.034167 | 28.12 | -590.171901 | 28.29 |
| m18 | -590.630432 | 30.50 | -590.629762 | 29.92 | -590.725932 | 30.08 | -590.043910 | 30.21 | -590.031799 | 29.60 | -590.169439 | 29.84 |
| m19 | -590.656877 | 13.90 | -590.655667 | 13.66 | -590.751934 | 13.77 | -590.070171 | 13.73 | -590.057510 | 13.47 | -590.195263 | 13.63 |
| m20 | -590.651368 | 17.36 | -590.650258 | 17.05 | -590.746708 | 17.05 | -590.064571 | 17.24 | -590.052000 | 16.92 | -590.189925 | 16.98 |
| m21 | -590.655796 | 14.58 | -590.654523 | 14.38 | -590.750727 | 14.52 | -590.068839 | 14.56 | -590.056153 | 14.32 | -590.193826 | 14.53 |
| m22 | -590.658068 | 13.16 | -590.656884 | 12.90 | -590.752970 | 13.12 | -590.071090 | 13.15 | -590.058486 | 12.85 | -590.196018 | 13.16 |
| m23 | -590.664799 | 8.93 | -590.663563 | 8.71 | -590.759702 | 8.89 | -590.078053 | 8.78 | -590.065406 | 8.51 | -590.203021 | 8.77 |
| m24 | -590.637935 | 25.79 | -590.637162 | 25.27 | -590.733459 | 25.36 | -590.051280 | 25.58 | -590.039090 | 25.03 | -590.176811 | 25.21 |
| m25 | -590.629774 | 30.91 | -590.629149 | 30.30 | -590.725407 | 30.41 | -590.043138 | 30.69 | -590.031080 | 30.05 | -590.168715 | 30.29 |
| m26 | -590.660404 | 11.69 | -590.659237 | 11.42 | -590.755778 | 11.35 | -590.073448 | 11.67 | -590.060771 | 11.42 | -590.198870 | 11.37 |
| m27 | -590.643844 | 22.08 | -590.643306 | 21.42 | -590.739214 | 21.75 | -590.057357 | 21.77 | -590.045470 | 21.02 | -590.182709 | 21.51 |
| m28 | -590.630909 | 30.20 | -590.630118 | 29.69 | -590.726415 | 29.78 | -590.044465 | 29.86 | -590.032264 | 29.31 | -590.169934 | 29.53 |
| m29 | -590.639163 | 25.02 | -590.638648 | 24.34 | -590.734808 | 24.51 | -590.052692 | 24.70 | -590.040799 | 23.95 | -590.178347 | 24.25 |
| m30 | -590.623422 | 34.90 | -590.622643 | 34.38 | -590.718860 | 34.52 | -590.036802 | 34.67 | -590.024634 | 34.10 | -590.162237 | 34.36 |
| m31 | -590.659343 | 12.36 | -590.657849 | 12.29 | -590.754050 | 12.44 | -590.072588 | 12.21 | -590.059679 | 12.11 | -590.197381 | 12.30 |
| m32 | -590.663108 | 9.99 | -590.662001 | 9.69 | -590.758413 | 9.70 | -590.076323 | 9.87 | -590.063761 | 9.54 | -590.201680 | 9.61 |
| m33 | -590.635051 | 27.60 | -590.634301 | 27.07 | -590.730744 | 27.06 | -590.048350 | 27.42 | -590.036148 | 26.87 | -590.174030 | 26.96 |
| m34 | -590.642154 | 23.14 | -590.641288 | 22.68 | -590.737423 | 22.87 | -590.055615 | 22.86 | -590.043334 | 22.36 | -590.180883 | 22.66 |
| m35 | -590.626396 | 33.03 | -590.625539 | 32.57 | -590.721851 | 32.64 | -590.039715 | 32.84 | -590.027390 | 32.37 | -590.165184 | 32.51 |
| m36 | -590.641533 | 23.53 | -590.641056 | 22.83 | -590.737143 | 23.05 | -590.055093 | 23.19 | -590.043251 | 22.41 | -590.180677 | 22.79 |
| m37 | -590.617420 | 38.66 | -590.616471 | 38.26 | -590.713074 | 38.15 | -590.030745 | 38.47 | -590.018364 | 38.03 | -590.156357 | 38.05 |
| m38 | -590.622969 | 35.18 | -590.622079 | 34.74 | -590.718488 | 34.75 | -590.036224 | 35.03 | -590.023854 | 34.59 | -590.161739 | 34.67 |
| m39 | -590.637993 | 25.76 | -590.637308 | 25.18 | -590.733438 | 25.37 | -590.051364 | 25.53 | -590.039289 | 24.90 | -590.176777 | 25.23 |
| m40 | -590.634691 | 27.83 | -590.633977 | 27.27 | -590.729907 | 27.59 | -590.048264 | 27.48 | -590.036207 | 26.83 | -590.173479 | 27.30 |
| m41 | -590.636627 | 26.61 | -590.636013 | 25.99 | -590.731854 | 26.37 | -590.050115 | 26.31 | -590.038153 | 25.61 | -590.175314 | 26.15 |
| m42 | -590.626592 | 32.91 | -590.625913 | 32.33 | -590.722145 | 32.46 | -590.040079 | 32.61 | -590.027972 | 32.00 | -590.165600 | 32.25 |
| m43 | -590.643576 | 22.25 | -590.642357 | 22.01 | -590.738347 | 22.29 | -590.057472 | 21.70 | -590.044902 | 21.38 | -590.182303 | 21.77 |
| m44 | -590.630075 | 30.72 | -590.629458 | 30.11 | -590.725649 | 30.26 | -590.043549 | 30.43 | -590.031529 | 29.77 | -590.169116 | 30.04 |
| m45 | -590.646071 | 20.69 | -590.644863 | 20.44 | -590.741100 | 20.56 | -590.059379 | 20.50 | -590.046711 | 20.24 | -590.184429 | 20.43 |
| ^a^ Units for *E*_e_ and Δ*E* in hartrees and kcal/mol, respectively. | | | | | | | | | | | | |

| **Table S4**  Absolute and relative electronic energies by MP2 and CCSD(T) methods of the 45 local minima of the cationic Ac-dAba-NHMe optimized at the SMD M06-2X/6-31+G(d) level of theory in water.^a^ | | | | | | | | | | |
| --- | --- | --- | --- | --- | --- | --- | --- | --- | --- | --- |
| Conf. | MP2 | | | | | | | | CCSD(T) | |
|  | cc-pVDZ | | aug-cc-pVDZ | | aug-cc-pVTZ | | aug-cc-pVQZ | | cc-pVDZ | |
|  | *E*_e_ | Δ*E* | *E*_e_ | Δ*E* | *E*_e_ | Δ*E* | *E*_e_ | Δ*E* | *E*_e_ | Δ*E* |
| m01 | -589.204264 | 0.00 | -589.328377 | 0.00 | -589.848918 | 0.00 | -590.014915 | 0.00 | -589.365750 | 0.00 |
| m02 | -589.181399 | 14.35 | -589.306773 | 13.56 | -589.827220 | 13.62 | -589.993216 | 13.62 | -589.342837 | 14.38 |
| m03 | -589.172474 | 19.95 | -589.299018 | 18.42 | -589.819177 | 18.66 | -589.985081 | 18.72 | -589.333371 | 20.32 |
| m04 | -589.186706 | 11.02 | -589.311963 | 10.30 | -589.832615 | 10.23 | -589.998650 | 10.21 | -589.348260 | 10.98 |
| m05 | -589.185838 | 11.56 | -589.311321 | 10.70 | -589.831483 | 10.94 | -589.997305 | 11.05 | -589.347260 | 11.60 |
| m06 | -589.164950 | 24.67 | -589.290893 | 23.52 | -589.811092 | 23.74 | -589.976942 | 23.83 | -589.325632 | 25.17 |
| m07 | -589.181512 | 14.28 | -589.307738 | 12.95 | -589.828114 | 13.05 | -589.994038 | 13.10 | -589.342531 | 14.57 |
| m08 | -589.182417 | 13.71 | -589.307927 | 12.83 | -589.828291 | 12.94 | -589.994292 | 12.94 | -589.343512 | 13.95 |
| m09 | -589.190754 | 8.48 | -589.315613 | 8.01 | -589.836231 | 7.96 | -590.002151 | 8.01 | -589.352103 | 8.56 |
| m10 | -589.180243 | 15.07 | -589.305673 | 14.25 | -589.826117 | 14.31 | -589.992023 | 14.36 | -589.341359 | 15.31 |
| m11 | -589.173955 | 19.02 | -589.300985 | 17.19 | -589.821270 | 17.35 | -589.987228 | 17.37 | -589.334956 | 19.32 |
| m12 | -589.183933 | 12.76 | -589.309798 | 11.66 | -589.830469 | 11.58 | -589.996506 | 11.55 | -589.345626 | 12.63 |
| m13 | -589.183993 | 12.72 | -589.309146 | 12.07 | -589.829320 | 12.30 | -589.995241 | 12.35 | -589.345377 | 12.78 |
| m14 | -589.158541 | 28.69 | -589.285210 | 27.09 | -589.804690 | 27.75 | -589.970354 | 27.96 | -589.319485 | 29.03 |
| m15 | -589.196809 | 4.68 | -589.321429 | 4.36 | -589.840950 | 5.00 | -590.006474 | 5.30 | -589.357899 | 4.93 |
| m16 | -589.196985 | 4.57 | -589.322163 | 3.90 | -589.842631 | 3.95 | -590.008639 | 3.94 | -589.358802 | 4.36 |
| m17 | -589.158203 | 28.90 | -589.284325 | 27.64 | -589.804421 | 27.92 | -589.970357 | 27.96 | -589.319342 | 29.12 |
| m18 | -589.155456 | 30.63 | -589.282604 | 28.72 | -589.802724 | 28.99 | -589.968410 | 29.18 | -589.316159 | 31.12 |
| m19 | -589.182536 | 13.63 | -589.307153 | 13.32 | -589.827549 | 13.41 | -589.993505 | 13.44 | -589.343695 | 13.84 |
| m20 | -589.176361 | 17.51 | -589.301726 | 16.72 | -589.822038 | 16.87 | -589.988044 | 16.86 | -589.337652 | 17.63 |
| m21 | -589.181259 | 14.44 | -589.306349 | 13.82 | -589.826186 | 14.26 | -589.991927 | 14.43 | -589.342772 | 14.42 |
| m22 | -589.182953 | 13.37 | -589.308721 | 12.33 | -589.828596 | 12.75 | -589.994298 | 12.94 | -589.344308 | 13.45 |
| m23 | -589.190190 | 8.83 | -589.314685 | 8.59 | -589.835232 | 8.59 | -590.001156 | 8.63 | -589.351565 | 8.90 |
| m24 | -589.163177 | 25.78 | -589.288962 | 24.73 | -589.809322 | 24.85 | -589.975247 | 24.89 | -589.324370 | 25.97 |
| m25 | -589.155546 | 30.57 | -589.281224 | 29.59 | -589.801335 | 29.86 | -589.967255 | 29.91 | -589.316557 | 30.87 |
| m26 | -589.184819 | 12.20 | -589.309935 | 11.57 | -589.830635 | 11.47 | -589.996736 | 11.41 | -589.346403 | 12.14 |
| m27 | -589.169096 | 22.07 | -589.295681 | 20.52 | -589.815621 | 20.89 | -589.981451 | 21.00 | -589.330128 | 22.35 |
| m28 | -589.156123 | 30.21 | -589.282658 | 28.69 | -589.802798 | 28.94 | -589.968687 | 29.01 | -589.316916 | 30.64 |
| m29 | -589.163512 | 25.57 | -589.291295 | 23.27 | -589.811371 | 23.56 | -589.977082 | 23.74 | -589.324427 | 25.93 |
| m30 | -589.149119 | 34.60 | -589.275769 | 33.01 | -589.795260 | 33.67 | -589.960944 | 33.87 | -589.310384 | 34.74 |
| m31 | -589.185478 | 11.79 | -589.310080 | 11.48 | -589.830054 | 11.84 | -589.995831 | 11.98 | -589.346836 | 11.87 |
| m32 | -589.188109 | 10.14 | -589.313031 | 9.63 | -589.833266 | 9.82 | -589.999331 | 9.78 | -589.350211 | 9.75 |
| m33 | -589.159931 | 27.82 | -589.286140 | 26.50 | -589.806352 | 26.71 | -589.972330 | 26.72 | -589.321308 | 27.89 |
| m34 | -589.167967 | 22.78 | -589.293446 | 21.92 | -589.813579 | 22.18 | -589.979520 | 22.21 | -589.328904 | 23.12 |
| m35 | -589.151556 | 33.07 | -589.278026 | 31.60 | -589.798134 | 31.87 | -589.963863 | 32.04 | -589.312607 | 33.35 |
| m36 | -589.166440 | 23.73 | -589.293276 | 22.03 | -589.813287 | 22.36 | -589.979236 | 22.39 | -589.327572 | 23.96 |
| m37 | -589.142553 | 38.72 | -589.268979 | 37.27 | -589.789018 | 37.59 | -589.954926 | 37.64 | -589.303529 | 39.04 |
| m38 | -589.147922 | 35.36 | -589.274641 | 33.72 | -589.794760 | 33.98 | -589.960420 | 34.20 | -589.308982 | 35.62 |
| m39 | -589.163118 | 25.82 | -589.289419 | 24.45 | -589.809201 | 24.92 | -589.975060 | 25.01 | -589.324645 | 25.79 |
| m40 | -589.160869 | 27.23 | -589.286873 | 26.04 | -589.806590 | 26.56 | -589.972320 | 26.73 | -589.321893 | 27.52 |
| m41 | -589.162422 | 26.26 | -589.288919 | 24.76 | -589.808276 | 25.50 | -589.973995 | 25.68 | -589.323608 | 26.44 |
| m42 | -589.151659 | 33.01 | -589.278591 | 31.24 | -589.798575 | 31.59 | -589.964307 | 31.76 | -589.312523 | 33.40 |
| m43 | -589.171234 | 20.73 | -589.297672 | 19.27 | -589.816577 | 20.29 | -589.981909 | 20.71 | -589.331739 | 21.34 |
| m44 | -589.155529 | 30.58 | -589.281892 | 29.17 | -589.801955 | 29.47 | -589.967741 | 29.60 | -589.316730 | 30.76 |
| m45 | -589.171635 | 20.47 | -589.296722 | 19.86 | -589.816997 | 20.03 | -589.982695 | 20.22 | -589.333006 | 20.55 |
| ^a^ Units for *E*_e_ and Δ*E* in hartrees and kcal/mol, respectively. | | | | | | | | | | |

| **Table S5**  Absolute and relative electronic energies by MP2/CBS limit and CCSD(T)/CBS limit methods of the 45 local minima of the cationic Ac-dAba-NHMe optimized at the SMD M06-2X/6-31+G(d) level of theory in water.^a^ | | | | | |
| --- | --- | --- | --- | --- | --- |
| Conf. | MP2/CBS limit^b^ | | ΔCCSD(T)^c^ | CCSD(T)/CBS limit^d^ | |
|  | *E*_e_ | Δ*E* | *E*_e_ | *E*_e_ | Δ*E* |
| m01 | -590.136048 | 0.00 | -0.161486 | -590.297534 | 0.00 |
| m02 | -590.114348 | 13.62 | -0.161438 | -590.275786 | 13.65 |
| m03 | -590.106147 | 18.76 | -0.160897 | -590.267044 | 19.13 |
| m04 | -590.119811 | 10.19 | -0.161554 | -590.281365 | 10.15 |
| m05 | -590.118311 | 11.13 | -0.161422 | -590.279732 | 11.17 |
| m06 | -590.097967 | 23.90 | -0.160682 | -590.258649 | 24.40 |
| m07 | -590.115117 | 13.13 | -0.161019 | -590.276136 | 13.43 |
| m08 | -590.115428 | 12.94 | -0.161095 | -590.276523 | 13.18 |
| m09 | -590.123228 | 8.04 | -0.161349 | -590.284576 | 8.13 |
| m10 | -590.113089 | 14.41 | -0.161116 | -590.274206 | 14.64 |
| m11 | -590.108333 | 17.39 | -0.161001 | -590.269334 | 17.70 |
| m12 | -590.117669 | 11.53 | -0.161693 | -590.279361 | 11.40 |
| m13 | -590.116318 | 12.38 | -0.161384 | -590.277701 | 12.45 |
| m14 | -590.091244 | 28.11 | -0.160944 | -590.252188 | 28.45 |
| m15 | -590.127262 | 5.51 | -0.161090 | -590.288352 | 5.76 |
| m16 | -590.129781 | 3.93 | -0.161817 | -590.291597 | 3.73 |
| m17 | -590.091446 | 27.99 | -0.161138 | -590.252585 | 28.21 |
| m18 | -590.089316 | 29.32 | -0.160703 | -590.250019 | 29.82 |
| m19 | -590.114607 | 13.45 | -0.161159 | -590.275766 | 13.66 |
| m20 | -590.109184 | 16.86 | -0.161291 | -590.270475 | 16.98 |
| m21 | -590.112873 | 14.54 | -0.161513 | -590.274386 | 14.53 |
| m22 | -590.115216 | 13.07 | -0.161355 | -590.276571 | 13.15 |
| m23 | -590.122236 | 8.67 | -0.161375 | -590.283610 | 8.74 |
| m24 | -590.096327 | 24.92 | -0.161192 | -590.257520 | 25.11 |
| m25 | -590.088332 | 29.94 | -0.161012 | -590.249343 | 30.24 |
| m26 | -590.117945 | 11.36 | -0.161584 | -590.279528 | 11.30 |
| m27 | -590.102461 | 21.08 | -0.161032 | -590.263494 | 21.36 |
| m28 | -590.089741 | 29.06 | -0.160792 | -590.250533 | 29.49 |
| m29 | -590.098006 | 23.87 | -0.160915 | -590.258921 | 24.23 |
| m30 | -590.081848 | 34.01 | -0.161265 | -590.243113 | 34.15 |
| m31 | -590.116804 | 12.08 | -0.161359 | -590.278162 | 12.16 |
| m32 | -590.120513 | 9.75 | -0.162102 | -590.282615 | 9.36 |
| m33 | -590.093448 | 26.73 | -0.161377 | -590.254825 | 26.80 |
| m34 | -590.100612 | 22.24 | -0.160936 | -590.261548 | 22.58 |
| m35 | -590.084800 | 32.16 | -0.161051 | -590.245851 | 32.43 |
| m36 | -590.100334 | 22.41 | -0.161133 | -590.261467 | 22.63 |
| m37 | -590.075993 | 37.68 | -0.160976 | -590.236969 | 38.00 |
| m38 | -590.081306 | 34.35 | -0.161060 | -590.242366 | 34.62 |
| m39 | -590.096093 | 25.07 | -0.161528 | -590.257621 | 25.05 |
| m40 | -590.093258 | 26.85 | -0.161025 | -590.254283 | 27.14 |
| m41 | -590.094924 | 25.81 | -0.161185 | -590.256110 | 25.99 |
| m42 | -590.085247 | 31.88 | -0.160863 | -590.246110 | 32.27 |
| m43 | -590.102556 | 21.02 | -0.160505 | -590.263061 | 21.63 |
| m44 | -590.088719 | 29.70 | -0.161201 | -590.249920 | 29.88 |
| m45 | -590.103609 | 20.36 | -0.161371 | -590.264980 | 20.43 |
| ^a^ Units for *E*_e_ and Δ*E* in hartrees and kcal/mol, respectively. ^b^ The MP2/CBS limit energies were obtained using the two-point extrapolation scheme with MP2/aug-cc-pVTZ and MP2/aug-cc-pVQZ energies. ^c^ The ΔCCSD(T) values were calculated by the difference of single-point energies at the CCSD(T)/cc-pVDZ and MP2/cc-pVDZ levels of theory. ^d^ The CCSD(T)/CBS limit values were calculated by the sum of the MP2/CBS limit energies and ΔCCSD(T) values. | | | | | |

**Table 6**

Cartesian coordinates of the 45 local minima of the cationic Ac-dAba-NHMe optimized at the SMD M06-2X/6-31+G(d) level of theory in water**:**

m01

6 3.935183 -1.157013 -0.21222

6 2.644186 -0.388 -0.186425

8 2.468346 0.58863 -0.941626

7 1.711245 -0.796702 0.687987

6 0.359147 -0.248886 0.806089

6 0.335619 1.247479 1.141189

6 -0.479005 -0.656725 -0.425271

7 0.356341 2.124264 -0.063741

6 -1.908715 -0.165859 -0.346209

8 -2.189977 1.037185 -0.531105

7 -2.847463 -1.07034 -0.066619

6 -4.252818 -0.711492 0.032908

1 4.056014 -1.596424 -1.206728

1 3.973494 -1.947192 0.539538

1 4.759912 -0.457804 -0.050064

1 1.917331 -1.619347 1.243562

1 -0.063824 -0.744803 1.682778

1 -0.578782 1.486344 1.6852

1 1.192859 1.507257 1.762754

1 0.478823 3.105916 0.203639

1 -0.544931 2.02461 -0.561452

1 -0.034114 -0.246366 -1.338155

1 -0.450379 -1.746614 -0.499256

1 -2.57445 -2.038502 0.052746

1 -4.623071 -0.332481 -0.923487

1 -4.399907 0.054329 0.798417

1 -4.814271 -1.603761 0.306363

1 1.135894 1.830735 -0.677768

m02

6 -1.608878 2.825287 -0.452737

6 -1.653149 1.40032 0.018183

8 -2.527959 1.013244 0.819199

7 -0.712135 0.567266 -0.453843

6 -0.537969 -0.815164 -0.002632

6 -1.588985 -1.763171 -0.581744

6 0.854276 -1.29017 -0.410117

7 -2.861556 -1.695554 0.201239

6 1.945512 -0.460955 0.239713

8 1.891418 -0.152755 1.443049

7 2.974938 -0.110333 -0.53856

6 4.119972 0.613238 -0.011636

1 -2.591289 3.096013 -0.847988

1 -0.84665 2.996247 -1.214656

1 -1.404294 3.464355 0.411768

1 -0.04681 0.934457 -1.1275

1 -0.611703 -0.823381 1.091701

1 -1.821517 -1.511008 -1.616711

1 -1.245355 -2.796751 -0.532251

1 -3.634685 -2.139981 -0.304273

1 -3.099147 -0.709411 0.412938

1 0.986407 -2.320808 -0.063418

1 0.951492 -1.289296 -1.501522

1 2.972557 -0.387396 -1.512798

1 4.60652 0.039657 0.782015

1 3.810637 1.581047 0.391616

1 4.827682 0.77374 -0.823872

1 -2.754921 -2.177363 1.100937

m03

6 -1.507298 2.72991 -0.68144

6 -1.49401 1.406572 0.031277

8 -2.079757 1.241976 1.113727

7 -0.8107 0.405015 -0.561843

6 -0.580409 -0.883816 0.079371

6 -1.628831 -1.911067 -0.325891

6 0.811104 -1.395296 -0.272545

7 -2.995695 -1.413986 0.01326

6 1.880708 -0.461691 0.259384

8 1.783615 0.061398 1.382898

7 2.936488 -0.248969 -0.532879

6 4.055062 0.576562 -0.107635

1 -2.535884 3.094843 -0.733689

1 -1.085241 2.669047 -1.68608

1 -0.923498 3.44212 -0.089773

1 -0.346293 0.590201 -1.446022

1 -0.641918 -0.723272 1.160068

1 -1.614203 -2.10659 -1.399746

1 -1.480639 -2.843748 0.219907

1 -3.703165 -2.133134 -0.175576

1 -3.242821 -0.583868 -0.538686

1 0.961668 -2.373959 0.19774

1 0.909494 -1.527235 -1.355918

1 2.970493 -0.699834 -1.439309

1 4.503647 0.178029 0.806333

1 3.727952 1.60282 0.079908

1 4.800773 0.578274 -0.901682

1 -3.059831 -1.152148 1.004646

m04

6 -1.963013 2.727926 -0.116979

6 -1.816444 1.261766 0.175513

8 -2.639492 0.670517 0.90509

7 -0.776966 0.627244 -0.383628

6 -0.418565 -0.762316 -0.094932

6 -1.474286 -1.764982 -0.575387

6 0.913131 -1.091645 -0.784361

7 -2.486832 -2.038158 0.485431

6 2.005385 -0.112193 -0.412975

8 2.04363 1.027412 -0.917073

7 2.903359 -0.518889 0.488023

6 3.978532 0.349135 0.938345

1 -2.939664 2.894722 -0.579945

1 -1.178604 3.108281 -0.773324

1 -1.941575 3.273962 0.830464

1 -0.090836 1.180719 -0.892666

1 -0.292115 -0.871883 0.990879

1 -2.003699 -1.38846 -1.451667

1 -1.010996 -2.720861 -0.821827

1 -3.264063 -2.597931 0.120058

1 -2.841648 -1.130009 0.841468

1 1.194933 -2.109703 -0.50551

1 0.779489 -1.051408 -1.871044

1 2.832062 -1.456579 0.864294

1 3.575051 1.264298 1.379502

1 4.630502 0.618049 0.102701

1 4.559828 -0.18412 1.689337

1 -2.067129 -2.550024 1.269021

m05

6 -0.968231 2.959425 0.370462

6 -1.184872 1.507861 0.048696

8 -1.729828 1.169516 -1.021415

7 -0.774833 0.610011 0.959591

6 -0.716369 -0.842164 0.754776

6 -2.073998 -1.504623 0.526672

6 0.317063 -1.212447 -0.314

7 -2.5326 -1.446327 -0.894484

6 1.68935 -0.719244 0.096446

8 2.139772 -0.937227 1.23494

7 2.384866 -0.043583 -0.823906

6 3.730589 0.439325 -0.559629

1 -1.920752 3.486062 0.269379

1 -0.273458 3.372705 -0.367177

1 -0.56452 3.117693 1.371665

1 -0.287695 0.963107 1.776348

1 -0.366973 -1.238947 1.710296

1 -2.010363 -2.559369 0.795472

1 -2.843577 -1.028113 1.134029

1 -3.516908 -1.723364 -0.967538

1 -2.414719 -0.474244 -1.242432

1 0.363902 -2.305333 -0.399588

1 0.044658 -0.807037 -1.293323

1 1.972942 0.109812 -1.736333

1 4.395164 -0.391298 -0.30703

1 3.727699 1.152891 0.268712

1 4.100025 0.934674 -1.456519

1 -1.988198 -2.077061 -1.493104

m06

6 -1.062697 2.808528 -0.791182

1 -0.157364 3.349811 -0.497349

6 -1.15059 1.557638 0.040791

8 -1.454416 1.600279 1.242181

7 -0.867105 0.395695 -0.590222

1 -0.593042 0.432801 -1.568311

6 -0.740989 -0.883563 0.094253

1 -0.81337 -0.679969 1.166037

6 -1.859425 -1.834224 -0.30766

1 -1.861375 -2.026451 -1.382363

1 -1.777477 -2.777004 0.234051

1 -3.34165 -0.359241 -0.472153

1 -3.950547 -1.877819 -0.201674

6 0.616125 -1.510816 -0.209845

1 0.711718 -1.71774 -1.281493

6 1.732617 -0.605252 0.268057

8 1.675986 -0.036081 1.372063

7 2.780824 -0.463464 -0.550107

1 2.780867 -0.952481 -1.436982

6 3.944167 0.320706 -0.169521

1 3.665217 1.362643 0.008979

1 -1.020637 2.603773 -1.862621

1 0.704095 -2.462629 0.326913

7 -3.183999 -1.237987 0.036838

1 -1.923573 3.443141 -0.569074

1 -3.247073 -1.028513 1.040882

1 4.40093 -0.080945 0.739214

1 4.667427 0.279218 -0.982925

m07

6 3.002316 -1.963487 -0.505775

6 2.435477 -0.698473 0.076751

8 3.174346 0.182904 0.550378

7 1.095346 -0.566553 0.059292

6 0.407469 0.552434 0.690817

6 0.168523 1.702367 -0.282499

6 -0.920568 0.105726 1.310554

7 1.461375 2.201495 -0.837649

6 -1.901746 -0.421391 0.282741

8 -1.572664 -1.319973 -0.517046

7 -3.127397 0.108908 0.278103

6 -4.155465 -0.359712 -0.636919

1 3.533801 -2.49704 0.287929

1 3.728052 -1.700341 -1.279919

1 2.235629 -2.615807 -0.927268

1 0.513623 -1.29128 -0.354488

1 1.049104 0.912251 1.500774

1 -0.445613 1.399022 -1.133585

1 -0.305878 2.539892 0.230855

1 2.114299 2.459409 -0.087487

1 1.924774 1.491356 -1.41644

1 -0.722361 -0.702866 2.02199

1 -1.347957 0.945395 1.865455

1 -3.351637 0.841884 0.940502

1 -3.858012 -0.184441 -1.674258

1 -5.073703 0.188491 -0.430342

1 -4.334839 -1.428855 -0.496109

1 1.305946 3.027749 -1.426502

m08

6 4.125891 -0.63572 -0.644003

6 2.858559 -0.539277 0.159701

8 2.850832 -0.72731 1.386443

7 1.72837 -0.24821 -0.516631

6 0.436276 -0.128783 0.145553

6 0.298289 1.213729 0.863862

6 -0.654418 -0.41877 -0.899762

7 0.026593 2.330416 -0.089694

6 -2.050421 -0.133795 -0.388362

8 -2.468398 1.039326 -0.292016

7 -2.801575 -1.180057 -0.047866

6 -4.153145 -1.021047 0.465483

1 4.847146 0.084053 -0.246963

1 3.967965 -0.443137 -1.706714

1 4.543623 -1.63818 -0.514731

1 1.784319 -0.065784 -1.513628

1 0.394917 -0.892233 0.928243

1 -0.533264 1.19425 1.569281

1 1.216713 1.449199 1.402604

1 0.076545 3.237198 0.387539

1 -0.928655 2.213524 -0.472575

1 -0.500404 0.215113 -1.782054

1 -0.558792 -1.460378 -1.215833

1 -2.416428 -2.111927 -0.14621

1 -4.780498 -0.504165 -0.265386

1 -4.147454 -0.446764 1.395554

1 -4.564895 -2.011308 0.654816

1 0.699793 2.351349 -0.864856

m09

6 -2.751659 2.017021 -0.826776

6 -2.378226 0.86214 0.060901

8 -3.233865 0.22918 0.697756

7 -1.066098 0.551418 0.136796

6 -0.578912 -0.586689 0.906794

6 -0.897334 -1.9212 0.216032

6 0.8816 -0.377482 1.318063

7 -0.632084 -1.876126 -1.24947

6 1.852923 -0.060512 0.199654

8 1.733156 -0.536236 -0.950122

7 2.873612 0.737195 0.511501

6 3.913613 1.064806 -0.450725

1 -3.459562 1.659599 -1.579957

1 -1.889421 2.466879 -1.32188

1 -3.258034 2.77353 -0.22064

1 -0.419791 1.052392 -0.465605

1 -1.149493 -0.612202 1.839679

1 -0.288784 -2.721891 0.638642

1 -1.951497 -2.168616 0.340714

1 -0.716979 -2.807746 -1.667962

1 0.326706 -1.501555 -1.405233

1 0.923165 0.416559 2.069513

1 1.23676 -1.29573 1.801211

1 2.930095 1.118809 1.448438

1 3.484941 1.566943 -1.321779

1 4.430125 0.159884 -0.781157

1 4.627214 1.730947 0.031964

1 -1.299802 -1.257267 -1.722469

m10

6 3.304206 -1.300062 -0.807959

6 2.510694 -0.332242 0.025609

8 2.875646 -0.006746 1.16571

7 1.38977 0.172672 -0.532713

6 0.486713 1.041803 0.203114

6 -0.214365 1.928919 -0.824584

6 -0.452628 0.244473 1.135231

7 -1.311975 2.711933 -0.19228

6 -1.571105 -0.462648 0.404042

8 -2.525982 0.189419 -0.07282

7 -1.501195 -1.788551 0.285708

6 -2.530525 -2.550091 -0.403671

1 4.319625 -0.909998 -0.920823

1 2.86735 -1.46562 -1.794613

1 3.365702 -2.251357 -0.271721

1 1.078605 -0.20437 -1.423076

1 1.102108 1.675992 0.84675

1 0.495416 2.633113 -1.258276

1 -0.670238 1.339326 -1.623191

1 -0.991272 3.208389 0.647405

1 -1.685098 3.407887 -0.846744

1 0.159997 -0.457661 1.707998

1 -0.918365 0.932123 1.849738

1 -0.712434 -2.275586 0.695133

1 -2.600745 -2.240248 -1.449612

1 -3.501553 -2.401126 0.075721

1 -2.264925 -3.605255 -0.358456

1 -2.075615 2.059716 0.06495

m11

6 3.932246 -1.241936 -0.329938

6 2.756273 -0.466744 0.194038

8 2.863293 0.310348 1.157181

7 1.581484 -0.636326 -0.44726

6 0.335091 -0.03875 0.014795

6 0.102368 1.339582 -0.595959

6 -0.825435 -0.967271 -0.344196

7 1.225577 2.266966 -0.268746

6 -2.128753 -0.484024 0.258279

8 -2.205947 -0.178597 1.461331

7 -3.183406 -0.417391 -0.560467

6 -4.494262 -0.00846 -0.08371

1 4.395948 -1.777682 0.502443

1 4.666521 -0.534121 -0.72591

1 3.654588 -1.949834 -1.112778

1 1.540516 -1.293789 -1.219359

1 0.401136 0.05984 1.102922

1 0.032737 1.286455 -1.684194

1 -0.806656 1.791258 -0.193679

1 1.01429 3.220275 -0.584775

1 1.404811 2.29335 0.742545

1 -0.90607 -1.066406 -1.432282

1 -0.625743 -1.959247 0.075425

1 -3.068481 -0.675674 -1.532801

1 -4.446608 0.993772 0.350021

1 -4.866367 -0.70511 0.672453

1 -5.17928 -0.000045 -0.93054

1 2.099146 1.975821 -0.722123

m12

6 0.211202 2.813318 -0.622074

6 0.605971 1.534845 0.066212

8 0.748392 1.474809 1.295787

7 0.774616 0.453336 -0.728942

6 0.947583 -0.912614 -0.234701

6 2.261684 -1.094648 0.515144

6 -0.214299 -1.371949 0.659145

7 3.409861 -0.649088 -0.326168

6 -1.528715 -1.044493 -0.013626

8 -1.829878 -1.539966 -1.11484

7 -2.332758 -0.186492 0.623156

6 -3.594057 0.252844 0.049969

1 -0.849938 2.992542 -0.417059

1 0.359611 2.772867 -1.702794

1 0.784054 3.641121 -0.198373

1 0.558663 0.559242 -1.713955

1 0.96686 -1.54088 -1.129643

1 2.290825 -0.509761 1.433897

1 2.41604 -2.149334 0.745504

1 3.336912 0.350874 -0.550647

1 4.299344 -0.795355 0.163992

1 -0.151322 -2.458135 0.7803

1 -0.147877 -0.910503 1.647351

1 -2.004932 0.243046 1.480473

1 -4.251275 -0.602152 -0.128152

1 -3.430596 0.774989 -0.897611

1 -4.072119 0.931798 0.755088

1 3.449371 -1.167205 -1.212692

m13

6 -1.612839 2.730799 -0.437402

6 -1.515172 1.358796 0.168896

8 -2.054397 1.088038 1.255053

7 -0.830688 0.428538 -0.529896

6 -0.492457 -0.874473 0.030425

6 -1.563761 -1.921084 -0.253316

6 0.833163 -1.378318 -0.555053

7 -2.887868 -1.504706 0.29839

6 1.933241 -0.347984 -0.417803

8 2.046723 0.576796 -1.243398

7 2.754271 -0.466809 0.630333

6 3.820565 0.49086 0.872264

1 -1.197228 3.451589 0.272475

1 -2.668884 2.973333 -0.585697

1 -1.08455 2.811079 -1.389085

1 -0.326332 0.729461 -1.359162

1 -0.393133 -0.75405 1.115315

1 -1.696668 -2.075249 -1.325715

1 -1.300986 -2.866438 0.223766

1 -3.568536 -2.268465 0.219513

1 -2.812881 -1.235696 1.286981

1 1.099696 -2.3106 -0.050067

1 0.706058 -1.582137 -1.623545

1 2.625421 -1.241074 1.270883

1 3.410628 1.493208 1.022823

1 4.512524 0.515874 0.026149

1 4.360323 0.186172 1.767843

1 -3.270509 -0.696417 -0.205576

m14

6 0.429812 3.002621 0.422049

6 0.385609 1.65095 -0.233743

8 -0.275339 1.448426 -1.261904

7 1.109717 0.67169 0.358429

6 0.981981 -0.727918 -0.018526

6 2.309495 -1.436063 0.198662

6 -0.119113 -1.445226 0.782101

7 3.368227 -0.817055 -0.652325

6 -1.450925 -0.752521 0.588326

8 -1.801631 0.195991 1.312308

7 -2.214501 -1.195066 -0.418974

6 -3.428958 -0.500537 -0.811071

1 0.623275 3.763127 -0.338222

1 1.183284 3.06463 1.20963

1 -0.556557 3.198787 0.855611

1 1.499007 0.867601 1.277474

1 0.729272 -0.754648 -1.083453

1 2.640715 -1.359391 1.236611

1 2.237055 -2.486271 -0.083619

1 4.264738 -1.302285 -0.535238

1 3.510192 0.170698 -0.408577

1 -0.173183 -2.486311 0.449566

1 0.129978 -1.422658 1.847318

1 -1.853895 -1.931481 -1.014124

1 -3.209134 0.535474 -1.088065

1 -4.152349 -0.500274 0.008289

1 -3.861448 -1.018426 -1.666417

1 3.11331 -0.858725 -1.646693

m15

6 1.329143 2.693645 -0.099613

6 0.26827 1.639823 -0.243155

8 0.11552 1.035101 -1.320282

7 -0.482138 1.385865 0.845281

6 -1.365537 0.232804 1.044818

6 -2.471095 0.063985 0.002245

6 -0.573912 -1.05302 1.38156

7 -2.060867 -0.591863 -1.272269

6 0.488364 -1.417023 0.364348

8 0.22557 -2.099036 -0.64517

7 1.721118 -0.956273 0.601779

6 2.776153 -1.057989 -0.393281

1 1.337619 3.320335 -0.993741

1 2.296629 2.181834 -0.031674

1 1.193151 3.311809 0.789445

1 -0.23772 1.890491 1.691132

1 -1.921208 0.477469 1.953134

1 -3.251884 -0.560836 0.437567

1 -2.898305 1.033672 -0.256499

1 -2.8603 -0.66159 -1.909001

1 -1.67005 -1.531836 -1.113066

1 -0.106707 -0.90556 2.359069

1 -1.276876 -1.888468 1.458305

1 1.867316 -0.355203 1.40503

1 2.469302 -0.578761 -1.32878

1 3.013023 -2.105339 -0.596988

1 3.664221 -0.560914 -0.004496

1 -1.30204 -0.033078 -1.700889

m16

6 -4.064505 -1.226797 -0.058637

6 -2.833099 -0.37512 -0.173732

8 -2.892969 0.819908 -0.523896

7 -1.650775 -0.943238 0.113662

6 -0.42442 -0.1728 -0.021533

6 -0.412067 1.02844 0.943384

6 0.787814 -1.068948 0.174396

7 -0.485291 2.284896 0.149613

6 2.057875 -0.297213 -0.138296

8 2.024379 0.834664 -0.657602

7 3.208422 -0.904399 0.164235

6 4.49699 -0.299408 -0.131455

1 -4.496218 -1.350458 -1.056559

1 -4.79678 -0.705161 0.562961

1 -3.857463 -2.210305 0.367517

1 -1.60918 -1.91977 0.385318

1 -0.395458 0.213952 -1.047392

1 -1.270802 1.013761 1.614171

1 0.500275 1.074785 1.538689

1 -1.338171 2.255528 -0.428769

1 -0.515757 3.116828 0.748025

1 0.831058 -1.454224 1.199956

1 0.732033 -1.930468 -0.501093

1 3.177385 -1.827389 0.582115

1 4.603992 0.654 0.392709

1 4.606626 -0.124795 -1.205297

1 5.278751 -0.980181 0.202822

1 0.340403 2.347597 -0.460519

m17

6 -1.62482 2.990354 -0.52635

6 -1.615541 1.618775 0.08977

8 -2.31674 1.342602 1.075867

7 -0.799055 0.706209 -0.4755

6 -0.696996 -0.660228 0.01378

6 -1.865992 -1.461917 -0.559532

6 0.662428 -1.234751 -0.375146

7 -1.986765 -2.798007 0.096504

6 1.790136 -0.429616 0.239592

8 1.773053 -0.10969 1.440981

7 2.803813 -0.102545 -0.569064

6 3.970111 0.615855 -0.082341

1 -2.657768 3.260217 -0.761599

1 -1.016854 3.048503 -1.430958

1 -1.246456 3.705328 0.210138

1 -0.264254 0.968522 -1.29758

1 -0.787543 -0.630135 1.104814

1 -2.804786 -0.938227 -0.376011

1 -1.745724 -1.632726 -1.630906

1 -2.82754 -3.285566 -0.233822

1 -2.061643 -2.702243 1.116689

1 0.765543 -2.256479 0.008188

1 0.754532 -1.275388 -1.466078

1 2.771302 -0.390161 -1.539708

1 4.466191 0.052152 0.712326

1 3.683466 1.596416 0.30685

1 4.661534 0.748878 -0.913413

1 -1.182415 -3.402807 -0.105933

m18

6 1.395529 2.69031 1.02269

6 0.991221 1.569892 0.106592

8 0.664583 1.783271 -1.073755

7 0.989705 0.326453 0.630229

6 0.686968 -0.880975 -0.127929

6 1.864334 -1.844652 -0.045018

6 -0.567643 -1.578482 0.391314

7 3.088066 -1.211319 -0.61538

6 -1.88913 -0.851265 0.239226

8 -2.902478 -1.319921 0.791376

7 -1.951404 0.254448 -0.510736

6 -3.208665 0.962141 -0.695629

1 2.316864 3.135782 0.63521

1 1.558597 2.359695 2.049863

1 0.615681 3.456055 1.001477

1 1.227929 0.211377 1.611946

1 0.559804 -0.586331 -1.174642

1 2.088838 -2.112003 0.989718

1 1.666067 -2.747401 -0.62279

1 3.891144 -1.847476 -0.553603

1 3.33327 -0.348317 -0.113978

1 -0.687243 -2.538345 -0.124812

1 -0.450003 -1.80456 1.457412

1 -1.113834 0.653743 -0.924937

1 -3.592766 1.327874 0.2608

1 -3.956652 0.305165 -1.146824

1 -3.02849 1.809075 -1.356948

1 2.953697 -0.970011 -1.60513

m19

6 3.219958 -1.916121 -0.501416

6 2.62869 -0.616325 -0.029533

8 3.343784 0.361278 0.24223

7 1.286383 -0.571179 0.093824

6 0.583143 0.624482 0.532841

6 0.427671 1.558096 -0.671367

6 -0.728527 0.253909 1.246321

7 -0.421356 2.736148 -0.335897

6 -1.870507 -0.112924 0.321644

8 -2.416426 0.75896 -0.387423

7 -2.277658 -1.382534 0.310324

6 -3.383154 -1.823301 -0.525823

1 3.779246 -1.731383 -1.422904

1 2.464759 -2.682944 -0.683476

1 3.924832 -2.27339 0.254553

1 0.742667 -1.382668 -0.183452

1 1.219147 1.117053 1.274263

1 1.405038 1.925649 -0.985414

1 -0.051996 1.048482 -1.507774

1 -0.386203 3.4357 -1.08518

1 -0.11473 3.191407 0.532226

1 -0.516669 -0.551854 1.954068

1 -1.065356 1.117616 1.829931

1 -1.802654 -2.053801 0.902506

1 -3.161578 -1.645924 -1.581382

1 -4.29745 -1.285603 -0.260902

1 -3.533043 -2.889489 -0.36215

1 -1.400095 2.415313 -0.227119

m20

6 3.331985 -1.781539 -0.419043

6 2.659356 -0.506192 0.010599

8 3.313337 0.521443 0.257398

7 1.318467 -0.542563 0.124021

6 0.534466 0.622464 0.502306

6 0.304651 1.477457 -0.74699

6 -0.754273 0.192491 1.213412

7 -0.282023 2.807646 -0.4004

6 -1.684518 -0.609297 0.326599

8 -1.277301 -1.609645 -0.296133

7 -2.955074 -0.203721 0.255037

6 -3.949789 -0.938443 -0.50999

1 4.05 -2.073903 0.352381

1 3.887556 -1.591126 -1.341709

1 2.624068 -2.596212 -0.583123

1 0.804256 -1.375662 -0.150669

1 1.131827 1.202232 1.213767

1 1.255791 1.674799 -1.242536

1 -0.375557 0.996221 -1.4532

1 0.305097 3.306215 0.279791

1 -0.35681 3.396247 -1.23821

1 -0.479528 -0.444256 2.061655

1 -1.26785 1.065929 1.626737

1 -3.239293 0.616068 0.777635

1 -4.010784 -1.972782 -0.161362

1 -3.698452 -0.938177 -1.573848

1 -4.915809 -0.455357 -0.369677

1 -1.223667 2.724201 0.000284

m21

6 -1.287107 2.852743 0.344677

6 -1.398998 1.373963 0.106853

8 -2.063759 0.930946 -0.849479

7 -0.75283 0.564298 0.966173

6 -0.469958 -0.854646 0.725525

6 -1.702696 -1.75767 0.748344

6 0.390994 -1.068174 -0.534717

7 -2.456126 -1.772165 -0.542356

6 1.632355 -0.202547 -0.49241

8 1.644196 0.933337 -0.999695

7 2.698767 -0.707885 0.138477

6 3.930176 0.04878 0.283658

1 -0.655663 3.271285 -0.446395

1 -0.845848 3.092815 1.313598

1 -2.2781 3.30475 0.263189

1 -0.156132 1.014477 1.652521

1 0.112985 -1.167937 1.595257

1 -1.394532 -2.784564 0.946945

1 -2.396096 -1.436755 1.525595

1 -3.374352 -2.211079 -0.421337

1 -1.954703 -2.299312 -1.265885

1 0.654861 -2.129578 -0.592549

1 -0.15923 -0.792706 -1.43857

1 2.634718 -1.635971 0.539693

1 3.752599 0.975104 0.837066

1 4.344757 0.298389 -0.696876

1 4.647079 -0.562829 0.83003

1 -2.573108 -0.794789 -0.876537

m22

6 0.867162 2.852246 -0.602172

6 0.965462 1.518666 0.088376

8 1.147189 1.434062 1.312038

7 0.847531 0.425598 -0.698144

6 0.785613 -0.951364 -0.208551

6 2.06008 -1.368999 0.515447

6 -0.428618 -1.202574 0.690256

7 3.251192 -1.103542 -0.342504

6 -1.69853 -0.819905 -0.039097

8 -1.912439 -1.200884 -1.204256

7 -2.571469 -0.055443 0.624697

6 -3.828834 0.361786 0.026689

1 1.733456 3.458075 -0.324298

1 -0.031973 3.360727 -0.240719

1 0.81705 2.762373 -1.688663

1 0.62598 0.572938 -1.676354

1 0.684785 -1.569432 -1.10524

1 2.208897 -0.823644 1.446302

1 2.0372 -2.440458 0.717012

1 3.348041 -0.100143 -0.540838

1 3.182475 -1.595052 -1.242262

1 -0.480313 -2.27399 0.916414

1 -0.33457 -0.659599 1.634136

1 -2.352066 0.23707 1.569321

1 -4.442662 -0.506004 -0.229444

1 -3.646992 0.944406 -0.880496

1 -4.363681 0.97861 0.747718

1 4.111592 -1.416726 0.121129

m23

6 -3.057341 -1.814198 0.718931

6 -2.522868 -0.677883 -0.108643

8 -3.278661 0.099817 -0.712798

7 -1.181678 -0.548948 -0.172023

6 -0.538825 0.536635 -0.901022

6 -0.656282 1.878809 -0.155883

6 0.881807 0.152447 -1.312017

7 -0.540289 1.711647 1.321818

6 1.845558 -0.145222 -0.179691

8 1.505404 -0.113874 1.022507

7 3.089622 -0.461457 -0.538321

6 4.112403 -0.781145 0.444762

1 -3.751339 -1.409906 1.46083

1 -2.269356 -2.375166 1.224909

1 -3.619467 -2.486551 0.064089

1 -0.604029 -1.147971 0.409292

1 -1.096783 0.671256 -1.832163

1 0.132083 2.562942 -0.473312

1 -1.625467 2.339831 -0.344935

1 -0.448948 2.6209 1.785906

1 -1.370775 1.246177 1.704792

1 0.837185 -0.731198 -1.958899

1 1.297656 0.964954 -1.917592

1 3.325723 -0.479783 -1.524115

1 3.83336 -1.669922 1.016776

1 4.254795 0.054607 1.134751

1 5.046067 -0.973226 -0.082055

1 0.285044 1.120167 1.535115

m24

6 3.902978 -1.077901 -0.598959

6 2.618658 -0.780872 0.125973

8 2.378835 -1.262569 1.242977

7 1.748567 0.041444 -0.501021

6 0.422266 0.330788 0.023016

6 0.026092 1.747024 -0.364902

6 -0.602992 -0.682602 -0.494539

7 0.953634 2.731886 0.265796

6 -1.963907 -0.482921 0.137932

8 -2.084024 -0.233005 1.35097

7 -3.023665 -0.603507 -0.668691

6 -4.381541 -0.483711 -0.164929

1 3.940573 -2.15103 -0.808847

1 4.741296 -0.833615 0.058878

1 3.999265 -0.52382 -1.534362

1 1.942633 0.301769 -1.464382

1 0.481012 0.256762 1.112891

1 0.075931 1.896862 -1.445747

1 -0.979118 1.98312 -0.012907

1 0.71082 3.692978 -0.000404

1 0.91309 2.667916 1.290099

1 -0.666055 -0.631548 -1.586432

1 -0.257723 -1.686672 -0.221004

1 -2.873162 -0.818186 -1.64699

1 -4.544113 0.50761 0.266393

1 -4.57753 -1.238403 0.601605

1 -5.070583 -0.629874 -0.995621

1 1.926097 2.563134 -0.018877

m25

6 -1.054056 3.138235 -0.62713

6 -1.233287 1.823482 0.081289

8 -1.76133 1.759319 1.20255

7 -0.791201 0.725847 -0.565988

6 -0.851202 -0.609787 0.004062

6 -2.105973 -1.303257 -0.523452

6 0.438616 -1.360107 -0.319447

7 -2.305272 -2.631741 0.129925

6 1.630308 -0.616799 0.251193

8 1.620677 -0.183207 1.416621

7 2.683987 -0.458999 -0.556224

6 3.891424 0.218092 -0.112596

1 -2.033307 3.612112 -0.737786

1 -0.591245 3.030224 -1.609675

1 -0.43319 3.78638 -0.00206

1 -0.373305 0.833584 -1.485158

1 -0.942239 -0.494093 1.088081

1 -2.984823 -0.70083 -0.290738

1 -2.051043 -1.473873 -1.600197

1 -3.227597 -3.015806 -0.106508

1 -2.253547 -2.551482 1.153092

1 0.427116 -2.351548 0.148178

1 0.53912 -1.499767 -1.401792

1 2.637366 -0.821122 -1.500874

1 4.342927 -0.309726 0.731765

1 3.666072 1.243878 0.191192

1 4.597844 0.236838 -0.941431

1 -1.602493 -3.318564 -0.166966

m26

6 3.829064 -1.473102 -0.090092

6 2.690606 -0.550386 0.237895

8 2.812581 0.344104 1.099096

7 1.54102 -0.737047 -0.427151

6 0.313224 0.015368 -0.163723

6 0.400666 1.468272 -0.640317

6 -0.833311 -0.728512 -0.866809

7 1.002362 2.343215 0.410792

6 -2.19239 -0.227768 -0.422094

8 -2.67482 0.832147 -0.858621

7 -2.838952 -0.981003 0.474586

6 -4.139929 -0.596986 0.996256

1 4.69994 -0.872061 -0.364637

1 3.590026 -2.161922 -0.90192

1 4.082346 -2.043726 0.80822

1 1.507163 -1.467751 -1.131005

1 0.137413 0.003305 0.920278

1 1.020045 1.548865 -1.534388

1 -0.591196 1.867261 -0.851371

1 1.201949 3.279295 0.043897

1 1.871639 1.910406 0.771947

1 -0.752693 -0.585391 -1.950063

1 -0.739596 -1.796081 -0.648885

1 -2.410161 -1.84038 0.79665

1 -4.873349 -0.528138 0.188301

1 -4.080858 0.369237 1.504478

1 -4.462074 -1.356711 1.707221

1 0.358729 2.446313 1.203204

m27

6 -2.289886 2.474612 -0.611381

6 -2.091289 1.144129 0.06011

8 -3.02061 0.56724 0.6481

7 -0.857357 0.601823 -0.017234

6 -0.497756 -0.650312 0.637866

6 -0.677244 -1.845845 -0.292901

6 0.914548 -0.577717 1.230331

7 -2.124849 -2.027058 -0.623752

6 2.005946 -0.336138 0.20685

8 2.187442 -1.115639 -0.74592

7 2.771627 0.745152 0.391569

6 3.87076 1.065629 -0.503511

1 -3.039298 2.359827 -1.399741

1 -1.368475 2.87131 -1.041379

1 -2.681685 3.180569 0.126008

1 -0.147776 1.098166 -0.547756

1 -1.1857 -0.770886 1.480628

1 -0.147957 -1.719528 -1.236227

1 -0.345616 -2.763416 0.19528

1 -2.261119 -2.873321 -1.188506

1 -2.695462 -2.11573 0.225848

1 0.929563 0.19644 2.001942

1 1.13103 -1.536981 1.713009

1 2.581598 1.352578 1.179251

1 3.503374 1.222715 -1.52124

1 4.606169 0.256463 -0.51445

1 4.346514 1.978653 -0.147868

1 -2.496247 -1.235246 -1.160498

m28

6 0.060655 3.108794 0.493829

6 0.081402 1.831849 -0.299566

8 -0.743121 1.614749 -1.198208

7 1.05076 0.9414 0.021846

6 1.039833 -0.420342 -0.491696

6 2.441068 -0.922038 -0.811079

6 0.2672 -1.372072 0.440913

7 3.30191 -1.105176 0.400066

6 -1.173249 -0.924303 0.572825

8 -1.521039 -0.100048 1.437072

7 -2.043581 -1.439121 -0.304775

6 -3.414941 -0.964008 -0.373994

1 -0.033388 3.953087 -0.193332

1 0.950065 3.233845 1.114319

1 -0.825789 3.094039 1.136563

1 1.560267 1.122055 0.882297

1 0.529671 -0.382556 -1.458437

1 2.38196 -1.89625 -1.297127

1 2.960485 -0.22078 -1.464324

1 2.90579 -1.787096 1.057577

1 4.226903 -1.454174 0.121669

1 0.321001 -2.387344 0.03501

1 0.703948 -1.364917 1.445097

1 -1.699267 -2.067475 -1.020959

1 -3.440638 0.107073 -0.598727

1 -3.928064 -1.136787 0.575441

1 -3.929732 -1.510326 -1.163428

1 3.454637 -0.226997 0.910084

m29

6 -1.510611 2.610701 -0.990629

6 -1.405482 1.39158 -0.118738

8 -1.835588 1.384209 1.045855

7 -0.826413 0.29826 -0.66126

6 -0.50895 -0.892852 0.119283

6 -1.602221 -1.951072 0.012071

6 0.809107 -1.498859 -0.350448

7 -2.917658 -1.388734 0.439415

6 2.067326 -0.666699 -0.191312

8 3.104954 -1.023922 -0.778394

7 2.049881 0.413452 0.597326

6 3.241038 1.227444 0.785311

1 -2.560011 2.911917 -1.048718

1 -1.118255 2.449736 -1.99623

1 -0.954541 3.421674 -0.510621

1 -0.455335 0.362725 -1.604595

1 -0.444004 -0.592448 1.170882

1 -1.72166 -2.30857 -1.012373

1 -1.382433 -2.790655 0.672363

1 -3.632545 -2.124351 0.473372

1 -3.251447 -0.667129 -0.209943

1 0.986515 -2.434666 0.192345

1 0.73858 -1.759408 -1.412932

1 1.190207 0.710091 1.042629

1 3.583961 1.634927 -0.169561

1 4.046072 0.632356 1.224065

1 2.992843 2.047561 1.457673

1 -2.854781 -0.958361 1.370356

m30

6 -3.847733 -1.373537 -0.366486

6 -2.613628 -0.655075 0.107731

8 -2.399433 -0.461641 1.315167

7 -1.7514 -0.244472 -0.843101

6 -0.501499 0.449804 -0.559077

6 -0.830492 1.875311 -0.100139

6 0.400017 -0.336077 0.415892

7 0.405236 2.598763 0.332205

6 1.859309 -0.259128 0.012455

8 2.345921 0.77273 -0.49638

7 2.605818 -1.33953 0.245898

6 4.027923 -1.367362 -0.056603

1 -4.727143 -0.852476 0.021729

1 -3.842572 -2.384576 0.051156

1 -3.910719 -1.433494 -1.454455

1 -1.981608 -0.422997 -1.813447

1 0.014175 0.520772 -1.519856

1 -1.265073 2.439726 -0.924883

1 -1.518236 1.873239 0.745491

1 1.224591 2.294848 -0.224966

1 0.284996 3.613367 0.250441

1 0.316953 0.051108 1.439622

1 0.082186 -1.380877 0.454977

1 2.166984 -2.163947 0.6386

1 4.551441 -0.575285 0.484721

1 4.196124 -1.231981 -1.128503

1 4.424205 -2.334422 0.249665

1 0.630822 2.391347 1.311763

m31

6 3.883652 -1.112871 0.258954

6 2.498132 -0.70302 -0.162663

8 2.083148 -0.914178 -1.312085

7 1.746234 -0.080274 0.770296

6 0.364462 0.348599 0.580077

6 0.221256 1.372613 -0.54337

6 -0.583655 -0.833825 0.338834

7 1.153848 2.517701 -0.33422

6 -2.029205 -0.387975 0.425535

8 -2.444803 0.256743 1.405573

7 -2.828086 -0.720676 -0.593751

6 -4.2386 -0.369935 -0.605426

1 4.008263 -2.179675 0.054455

1 4.081044 -0.919625 1.314845

1 4.608753 -0.565756 -0.350911

1 2.137546 0.023609 1.699178

1 0.079332 0.831321 1.519699

1 0.446272 0.947084 -1.520781

1 -0.789577 1.784272 -0.547787

1 1.003621 3.244308 -1.043236

1 1.02026 2.947572 0.589003

1 -0.373182 -1.309725 -0.622451

1 -0.423532 -1.572066 1.13163

1 -2.441307 -1.239939 -1.372551

1 -4.670429 -0.717532 -1.543001

1 -4.365329 0.713461 -0.531684

1 -4.762691 -0.844333 0.228701

1 2.133969 2.217727 -0.400944

m32

6 -3.955384 -1.246407 -0.314958

6 -2.680608 -0.587359 0.136425

8 -2.42993 -0.413602 1.340969

7 -1.826054 -0.191879 -0.824886

6 -0.512283 0.377515 -0.53901

6 -0.652103 1.625069 0.353272

6 0.414587 -0.703305 0.039589

7 0.262179 2.7048 -0.119226

6 1.880333 -0.331488 0.016819

8 2.276586 0.852982 0.031267

7 2.746828 -1.346288 0.00917

6 4.183926 -1.125697 0.041041

1 -4.798244 -0.752842 0.175437

1 -3.93819 -2.290561 0.011942

1 -4.089961 -1.211635 -1.397544

1 -2.047899 -0.402666 -1.791131

1 -0.120589 0.690934 -1.512419

1 -1.665883 2.022382 0.310689

1 -0.38799 1.42077 1.39103

1 0.023295 3.005399 -1.070564

1 1.227047 2.324625 -0.120134

1 0.140404 -0.907062 1.081718

1 0.272426 -1.631293 -0.521865

1 2.391874 -2.294277 -0.029992

1 4.469659 -0.575827 0.941565

1 4.50523 -0.558184 -0.836449

1 4.679534 -2.095528 0.040847

1 0.214565 3.524321 0.494513

m33

6 2.175578 -2.295498 -0.340531

6 1.502173 -0.9536 -0.37612

8 0.81878 -0.612919 -1.362459

7 1.674104 -0.159479 0.69199

6 0.947195 1.080632 0.963776

6 1.108287 2.149921 -0.118596

6 -0.508593 0.851843 1.44879

7 0.183952 2.020903 -1.2833

6 -1.289058 -0.250197 0.754759

8 -1.023292 -1.447768 0.956268

7 -2.297378 0.115996 -0.04818

6 -3.092901 -0.870815 -0.759027

1 1.396863 -3.056491 -0.222109

1 2.891846 -2.389077 0.477473

1 2.676976 -2.467706 -1.295752

1 2.191387 -0.547905 1.473016

1 1.47373 1.515911 1.815526

1 0.920379 3.128751 0.323563

1 2.12489 2.127603 -0.512612

1 0.48449 2.636936 -2.046086

1 0.20257 1.034176 -1.610148

1 -0.460262 0.556345 2.500563

1 -1.04666 1.80469 1.396889

1 -2.531061 1.097587 -0.139508

1 -3.620772 -1.522803 -0.056867

1 -2.452402 -1.486288 -1.396978

1 -3.819977 -0.346173 -1.377826

1 -0.776732 2.284292 -1.041495

m34

6 4.133614 -0.770504 -0.449237

6 2.772379 -0.591533 0.166407

8 2.402449 -1.275944 1.133627

7 1.979808 0.350236 -0.382999

6 0.611788 0.574327 0.059089

6 0.307408 2.04179 -0.234947

6 -0.335712 -0.425547 -0.612162

7 -1.068424 2.429563 0.201145

6 -1.695942 -0.513072 0.053136

8 -1.877025 -0.144933 1.229279

7 -2.684892 -1.020198 -0.688426

6 -4.019165 -1.223401 -0.147705

1 4.206044 -1.789089 -0.841689

1 4.88896 -0.656674 0.333044

1 4.333001 -0.059541 -1.253435

1 2.298433 0.820599 -1.224479

1 0.589528 0.4167 1.141186

1 1.002914 2.679731 0.310538

1 0.377007 2.257802 -1.303499

1 -1.290823 2.045727 1.129472

1 -1.153561 3.451517 0.240615

1 -0.440943 -0.200688 -1.679779

1 0.113407 -1.423386 -0.53229

1 -2.484639 -1.307568 -1.639521

1 -4.44359 -0.274401 0.190075

1 -3.994438 -1.919758 0.695021

1 -4.648846 -1.636419 -0.93456

1 -1.787507 2.09291 -0.449474

m35

6 0.267988 3.272174 -0.791274

6 -0.032798 2.052667 0.033441

8 0.335649 1.964346 1.217812

7 -0.695944 1.053865 -0.582601

6 -1.092085 -0.185668 0.069509

6 -2.607666 -0.312059 -0.096876

6 -0.343988 -1.384696 -0.513702

7 -3.144972 -1.49576 0.639218

6 1.165592 -1.412903 -0.361318

8 1.827169 -2.216675 -1.044926

7 1.749074 -0.616512 0.540318

6 3.191294 -0.631279 0.728354

1 -0.067574 4.156611 -0.243671

1 -0.208974 3.244654 -1.772268

1 1.352437 3.343925 -0.918655

1 -0.938812 1.164604 -1.563102

1 -0.879361 -0.072172 1.137026

1 -3.101067 0.567274 0.316712

1 -2.887122 -0.429928 -1.146084

1 -2.833094 -1.499168 1.618694

1 -4.171211 -1.480065 0.63995

1 -0.691288 -2.315116 -0.047061

1 -0.559984 -1.466366 -1.584922

1 1.213738 0.096562 1.029015

1 3.709006 -0.35969 -0.19622

1 3.526903 -1.622971 1.042177

1 3.439449 0.092937 1.503793

1 -2.852723 -2.383017 0.214175

m36

6 2.280013 -2.690403 -0.195781

6 1.926473 -1.304955 0.270637

8 2.456133 -0.802871 1.275525

7 1.015135 -0.630102 -0.458703

6 0.52705 0.680578 -0.056357

6 1.594238 1.719711 -0.410069

6 -0.806509 0.979323 -0.752045

7 1.336995 3.024947 0.269528

6 -1.855003 -0.066248 -0.434185

8 -1.83 -1.186602 -0.976442

7 -2.791377 0.269317 0.458798

6 -3.845173 -0.654775 0.843966

1 2.075763 -3.393343 0.616677

1 3.352241 -2.723241 -0.408915

1 1.724451 -2.992028 -1.085783

1 0.512671 -1.122568 -1.191154

1 0.385605 0.675029 1.032529

1 2.574731 1.382431 -0.075548

1 1.619091 1.910507 -1.484521

1 1.241953 2.901742 1.285141

1 2.11686 3.672189 0.104928

1 -1.16165 1.969234 -0.451423

1 -0.652786 0.981874 -1.836639

1 -2.782432 1.201995 0.853219

1 -3.422476 -1.562307 1.282806

1 -4.449723 -0.931237 -0.024258

1 -4.479016 -0.163253 1.580938

1 0.483853 3.480243 -0.075021

m37

6 4.110773 -0.329635 -0.58566

6 2.739104 -0.591888 -0.026987

8 2.411163 -1.710922 0.397712

7 1.879489 0.447631 -0.019979

6 0.505369 0.299499 0.436747

6 -0.061161 1.615239 0.953076

6 -0.367971 -0.376179 -0.632706

7 -0.348839 2.625637 -0.11686

6 -1.72717 -0.733256 -0.069786

8 -1.827284 -1.428971 0.956686

7 -2.802439 -0.271155 -0.718001

6 -4.149345 -0.568516 -0.259741

1 4.855682 -0.728423 0.107062

1 4.299557 0.731683 -0.758036

1 4.206293 -0.868316 -1.533641

1 2.193203 1.323119 -0.425646

1 0.531094 -0.356704 1.312162

1 -1.009744 1.432359 1.460907

1 0.629512 2.08511 1.653803

1 -0.719863 3.482706 0.31146

1 -1.053076 2.293831 -0.7864

1 -0.449507 0.244269 -1.531286

1 0.120404 -1.31237 -0.922876

1 -2.670853 0.318065 -1.531495

1 -4.312807 -0.16668 0.744011

1 -4.316002 -1.648701 -0.239436

1 -4.856473 -0.110796 -0.950282

1 0.486478 2.893618 -0.651268

m38

6 -0.87477 3.041682 -0.978149

6 -0.597262 1.928732 -0.008597

8 0.007732 2.133937 1.058149

7 -1.031215 0.700653 -0.357499

6 -0.882714 -0.481502 0.48382

6 -2.233916 -1.083707 0.859882

6 0.053201 -1.516193 -0.144097

7 -2.950785 -1.716341 -0.293003

6 1.530572 -1.17794 -0.226117

8 2.293296 -1.963804 -0.819954

7 1.988151 -0.063824 0.353143

6 3.396967 0.290904 0.282648

1 0.069018 3.534983 -1.225398

1 -1.520331 3.775524 -0.486589

1 -1.356311 2.691998 -1.89294

1 -1.454224 0.589696 -1.274812

1 -0.477816 -0.137898 1.439477

1 -2.092727 -1.868722 1.603295

1 -2.897095 -0.318794 1.263621

1 -2.407452 -2.479604 -0.71382

1 -3.838168 -2.120128 0.030317

1 -0.010782 -2.452807 0.423083

1 -0.258505 -1.74545 -1.170736

1 1.353731 0.595555 0.795774

1 4.009749 -0.454969 0.796004

1 3.52926 1.25837 0.766053

1 3.726279 0.360125 -0.757671

1 -3.178375 -1.044214 -1.036044

m39

6 -0.239425 3.290658 -0.19924

6 -0.429591 1.809108 -0.376998

8 0.074912 1.201307 -1.333071

7 -1.16869 1.182704 0.561325

6 -1.314044 -0.264255 0.672469

6 -1.676665 -0.886815 -0.680475

6 -0.106876 -0.924227 1.375276

7 -2.42538 -2.165871 -0.468935

6 1.229763 -0.572818 0.756756

8 1.845178 0.453945 1.095349

7 1.721948 -1.421851 -0.155983

6 2.929674 -1.111778 -0.901915

1 0.799674 3.462903 0.101257

1 -0.902831 3.717849 0.555154

1 -0.401422 3.787235 -1.158778

1 -1.485041 1.727561 1.355626

1 -2.173386 -0.410227 1.334719

1 -2.341788 -0.229007 -1.240271

1 -0.811045 -1.128908 -1.293944

1 -3.281937 -2.013588 0.076647

1 -2.696721 -2.57196 -1.371857

1 -0.086725 -0.57468 2.41057

1 -0.247507 -2.010495 1.393634

1 1.163453 -2.221729 -0.429928

1 3.784718 -1.023834 -0.226511

1 2.813474 -0.169907 -1.447828

1 3.114799 -1.918963 -1.609851

1 -1.860597 -2.868394 0.023057

m40

6 1.333223 2.722933 -0.675804

6 1.292049 1.391558 0.023897

8 1.645468 1.262951 1.204018

7 0.835817 0.348034 -0.70744

6 0.533782 -0.972596 -0.160835

6 1.779942 -1.674216 0.370535

6 -0.540903 -0.950975 0.941928

7 2.848546 -1.684131 -0.67024

6 -1.74874 -0.153538 0.501324

8 -1.855391 1.058792 0.75988

7 -2.675536 -0.80865 -0.208736

6 -3.843538 -0.133828 -0.749522

1 0.455515 3.297681 -0.360241

1 1.314534 2.623939 -1.763015

1 2.228601 3.265503 -0.3664

1 0.4817 0.54872 -1.63635

1 0.154805 -1.553101 -1.00799

1 2.190225 -1.17624 1.247879

1 1.549905 -2.712998 0.609029

1 3.682317 -2.177795 -0.3325

1 3.128004 -0.72799 -0.921767

1 -0.818153 -1.986194 1.163672

1 -0.144922 -0.490538 1.849331

1 -2.536538 -1.793597 -0.40138

1 -3.54672 0.648074 -1.454391

1 -4.429798 0.321137 0.0532

1 -4.456339 -0.870418 -1.267665

1 2.529567 -2.15292 -1.526829

m41

6 0.267988 3.272174 -0.791274

6 -0.032798 2.052667 0.033441

8 0.335649 1.964346 1.217812

7 -0.695944 1.053865 -0.582601

6 -1.092085 -0.185668 0.069509

6 -2.607666 -0.312059 -0.096876

6 -0.343988 -1.384696 -0.513702

7 -3.144972 -1.49576 0.639218

6 1.165592 -1.412903 -0.361318

8 1.827169 -2.216675 -1.044926

7 1.749074 -0.616512 0.540318

6 3.191294 -0.631279 0.728354

1 -0.067574 4.156611 -0.243671

1 -0.208974 3.244654 -1.772268

1 1.352437 3.343925 -0.918655

1 -0.938812 1.164604 -1.563102

1 -0.879361 -0.072172 1.137026

1 -3.101067 0.567274 0.316712

1 -2.887122 -0.429928 -1.146084

1 -2.833094 -1.499168 1.618694

1 -4.171211 -1.480065 0.63995

1 -0.691288 -2.315116 -0.047061

1 -0.559984 -1.466366 -1.584922

1 1.213738 0.096562 1.029015

1 3.709006 -0.35969 -0.19622

1 3.526903 -1.622971 1.042177

1 3.439449 0.092937 1.503793

1 -2.852723 -2.383017 0.214175

m42

6 -1.847289 2.538664 -1.068487

6 -1.533419 1.514997 -0.012547

8 -1.804085 1.707121 1.182251

7 -0.935349 0.377281 -0.428528

6 -0.534424 -0.682472 0.487465

6 -1.6981 -1.624571 0.788871

6 0.686371 -1.415544 -0.060754

7 -2.187635 -2.3171 -0.441643

6 1.99066 -0.644193 -0.150986

8 2.969416 -1.181516 -0.700345

7 2.07401 0.578916 0.382561

6 3.307987 1.347139 0.318293

1 -2.925986 2.71885 -1.071976

1 -1.527039 2.229768 -2.065074

1 -1.350271 3.475372 -0.800647

1 -0.714188 0.272789 -1.414614

1 -0.295614 -0.222787 1.453046

1 -1.398042 -2.3962 1.498537

1 -2.542552 -1.063303 1.19057

1 -3.014325 -2.886891 -0.227545

1 -1.479667 -2.944599 -0.840167

1 0.891387 -2.288449 0.569481

1 0.494628 -1.794279 -1.072542

1 1.253869 1.01855 0.781253

1 3.601414 1.520645 -0.720685

1 4.115687 0.818566 0.830437

1 3.139195 2.305335 0.808199

1 -2.459039 -1.646011 -1.171393

m43

6 0.329104 2.807027 0.301731

6 -0.288802 1.484654 -0.05604

8 -0.304532 1.069494 -1.224944

7 -0.83006 0.774187 0.960611

6 -1.160114 -0.652584 0.920679

6 -1.919995 -1.085756 -0.33054

6 0.089802 -1.534291 1.130768

7 -2.991759 -0.105604 -0.669846

6 1.162119 -1.252134 0.10001

8 1.17069 -1.809024 -1.011807

7 2.090552 -0.348672 0.448478

6 3.043534 0.172728 -0.516366

1 0.064466 3.545522 -0.457945

1 1.418131 2.678383 0.288121

1 0.027645 3.160699 1.289328

1 -0.716305 1.158079 1.893436

1 -1.818753 -0.817859 1.777512

1 -1.284118 -1.175265 -1.207899

1 -2.403309 -2.043117 -0.132791

1 -3.598317 -0.47495 -1.410432

1 -2.577611 0.770314 -1.014286

1 0.470415 -1.348849 2.1391

1 -0.199759 -2.586472 1.055376

1 1.982779 0.134014 1.333615

1 2.525498 0.650026 -1.355522

1 3.674552 -0.630743 -0.904753

1 3.673783 0.907428 -0.016033

1 -3.583386 0.1118 0.141022

m44

6 3.188461 -1.942657 -0.235615

6 1.863328 -1.28541 0.028772

8 0.903862 -1.915105 0.507795

7 1.75173 0.024759 -0.268184

6 0.475803 0.702363 -0.096662

6 0.653813 2.208902 -0.14823

6 -0.557836 0.258618 -1.161448

7 1.420921 2.710847 1.032863

6 -1.934252 0.094519 -0.5505

8 -2.840824 0.929301 -0.718503

7 -2.098544 -1.002907 0.199483

6 -3.324822 -1.248234 0.939664

1 3.57288 -2.343695 0.706446

1 3.920669 -1.255252 -0.663177

1 3.032121 -2.78055 -0.920795

1 2.514129 0.493382 -0.749249

1 0.106145 0.428153 0.89787

1 1.189554 2.524214 -1.046149

1 -0.322611 2.694151 -0.12191

1 1.481418 3.735307 1.014656

1 0.964557 2.438351 1.912038

1 -0.617014 0.989708 -1.972327

1 -0.237435 -0.697393 -1.583517

1 -1.282222 -1.588343 0.3625

1 -4.175832 -1.324044 0.257897

1 -3.519123 -0.443502 1.655418

1 -3.218511 -2.188958 1.478865

1 2.381256 2.34741 1.052073

m45

6 -2.6604 -2.324503 -0.160026

6 -2.193584 -0.897173 -0.139448

8 -2.941278 0.018885 0.250467

7 -0.933011 -0.652977 -0.540646

6 -0.388476 0.700322 -0.672045

6 -0.390482 1.479102 0.656721

6 1.007779 0.645365 -1.292607

7 -1.579 2.375196 0.743292

6 2.166411 0.090386 -0.481022

8 3.328933 0.335961 -0.848577

7 1.923484 -0.67196 0.592656

6 3.00996 -1.248242 1.370711

1 -2.907129 -2.620458 0.86383

1 -1.915454 -3.010861 -0.566083

1 -3.57508 -2.381384 -0.75638

1 -0.410621 -1.419033 -0.955695

1 -1.018424 1.248527 -1.385451

1 -0.430915 0.816351 1.522062

1 0.491988 2.116021 0.734933

1 -1.550065 3.099604 0.017588

1 -1.626391 2.840037 1.65631

1 0.971585 0.067924 -2.224198

1 1.292576 1.664332 -1.568343

1 0.971745 -0.888738 0.862099

1 3.643533 -0.461218 1.787772

1 3.623963 -1.903021 0.746641

1 2.575725 -1.827783 2.184279

1 -2.425836 1.797585 0.604707

**Table 7**

Cartesian coordinates of the *H*_14_-helical structure of hexamer built from conformer m03 optimized at the SMD M06-2X/6-31G(d) level of theory in water**:**

6 -4.706481 -2.660537 -1.280665

6 -5.256858 -1.679777 -0.27763

8 -6.473662 -1.49588 -0.138963

7 -4.347543 -1.00259 0.457238

6 -4.745631 0.080823 1.345564

6 -4.991834 -0.41031 2.765049

6 -3.695722 1.185917 1.351516

7 -6.001326 -1.514606 2.75035

6 -3.553913 1.760837 -0.043681

8 -4.544372 2.005045 -0.73716

1 -5.465154 -3.413493 -1.496333

1 -3.798812 -3.144718 -0.911173

1 -4.464125 -2.123849 -2.204032

1 -3.35224 -1.202881 0.359769

1 -5.681048 0.488272 0.952365

1 -4.084569 -0.808098 3.225528

1 -5.395977 0.392913 3.381718

1 -6.366469 -1.695231 3.691906

1 -5.586224 -2.386489 2.40148

1 -4.031937 1.990597 2.014709

1 -2.740649 0.808168 1.725839

1 -6.785437 -1.288367 2.122714

7 -2.291719 2.004452 -0.45904

6 -2.006309 2.409315 -1.828454

1 -1.501246 1.751045 0.133772

6 -1.540404 3.855309 -1.884044

6 -0.963224 1.488538 -2.458012

1 -2.943219 2.310474 -2.382451

7 -2.600174 4.742207 -1.317346

1 -0.640305 4.003777 -1.284215

1 -1.360732 4.17433 -2.9109

6 -1.409343 0.05241 -2.282743

1 -0.889053 1.706862 -3.528483

1 0.019577 1.644263 -2.003301

1 -2.321902 5.729252 -1.359748

1 -2.784173 4.508015 -0.333408

1 -3.484135 4.637131 -1.831459

8 -2.569344 -0.303815 -2.517214

7 -0.461242 -0.802148 -1.851836

6 -0.7925 -2.146995 -1.403709

1 0.463865 -0.438273 -1.625432

6 -0.586313 -3.192131 -2.488424

6 0.029035 -2.467262 -0.16043

1 -1.850693 -2.142703 -1.128616

7 -1.5055 -2.918324 -3.635978

1 0.433432 -3.184651 -2.877353

1 -0.830969 -4.186989 -2.114715

6 -0.350334 -1.487868 0.931668

1 -0.212021 -3.476109 0.190209

1 1.099331 -2.424254 -0.383857

1 -1.412917 -3.642487 -4.357424

1 -1.305678 -2.011671 -4.074955

1 -2.485772 -2.896801 -3.325584

8 -1.540717 -1.25244 1.188066

7 0.651143 -0.904082 1.607733

6 0.389635 0.155587 2.577151

1 1.622343 -1.108498 1.363994

6 0.299172 -0.380681 3.997644

6 1.467236 1.229445 2.496627

1 -0.570668 0.605296 2.308014

7 -0.830942 -1.352826 4.115659

1 1.210683 -0.905548 4.288928

1 0.099067 0.428277 4.70049

6 1.501633 1.830367 1.106905

1 1.220989 2.03165 3.201071

1 2.443445 0.820495 2.769657

1 -0.946667 -1.657679 5.089261

1 -0.667081 -2.19068 3.545533

1 -1.715763 -0.935438 3.799553

8 0.46651 2.043474 0.466894

7 2.724721 2.152397 0.637346

6 2.942212 2.562434 -0.743907

1 3.534843 1.850415 1.173538

6 3.415103 4.006337 -0.813858

6 3.953341 1.64378 -1.421909

1 1.97862 2.477986 -1.25272

7 2.380248 4.902892 -0.217885

1 4.335436 4.155854 -0.247156

1 3.564088 4.320695 -1.846996

6 3.525392 0.195572 -1.283763

1 4.009048 1.890755 -2.487953

1 4.950108 1.788788 -0.992442

1 2.685534 5.882952 -0.237078

1 2.195259 4.650304 0.761621

1 1.492385 4.837603 -0.731024

8 2.337315 -0.13851 -1.271215

7 4.53214 -0.69878 -1.188995

6 4.323222 -2.080562 -0.776742

1 5.481099 -0.339749 -1.131279

6 4.677365 -3.050973 -1.890371

6 5.130532 -2.363679 0.485817

1 3.261504 -2.181312 -0.539053

7 3.786748 -2.802004 -3.063062

1 5.705676 -2.920771 -2.230829

1 4.520552 -4.082355 -1.574439

6 4.717883 -1.379159 1.562177

1 4.913171 -3.37765 0.838567

1 6.204281 -2.296376 0.28186

1 4.01219 -3.431428 -3.842032

1 3.888252 -1.836391 -3.40067

1 2.798973 -2.942266 -2.814471

8 3.524472 -1.09013 1.752654

7 5.696428 -0.833505 2.286605

6 5.411787 0.1361 3.330213

1 6.657068 -1.077654 2.081564

1 4.692771 -0.271554 4.044856

1 5.001017 1.056326 2.902303

1 6.340452 0.369378 3.848815
